# Variáveis Clínicas e Laboratoriais na Admissão Hospitalar são Preditores de Fibrilação Atrial Nova em Pacientes Internados com Pneumonia por COVID-19

**DOI:** 10.36660/abc.20220784

**Published:** 2024-03-20

**Authors:** Bruno Rustum Andrea, Paulo Roberto Benchimol-Barbosa, Simone Farah, Alexandra Monteiro

**Affiliations:** 1 Hospital Universitário Pedro Ernesto Faculdade de Ciências Médicas Telemedicina e Telessaúde Rio de Janeiro RJ Brasil Hospital Universitário Pedro Ernesto - Telemedicina e Telessaúde da Faculdade de Ciências Médicas, Rio de Janeiro, RJ – Brasil; 2 Hospital Universitário Pedro Ernesto Coordenadoria de Medicina Clínica Rio de Janeiro RJ Brasil Hospital Universitário Pedro Ernesto - Coordenadoria de Medicina Clínica, Rio de Janeiro, RJ – Brasil

**Keywords:** COVID-19, Fibrilação Atrial, Prognóstico, Hospitalização, Valor Preditivo dos Teses

## Abstract

**Fundamento:**

Fibrilação atrial nova (FAN) ocorre em pacientes internados por COVID-19. Há controvérsias quanto ao valor preditivo de dados clínicos e laboratoriais à admissão hospitalar para ocorrência de FAN.

**Objetivos:**

Analisar, à admissão hospitalar, variáveis com potencial preditivo para ocorrência de FAN em pacientes com pneumonia por COVID-19.

**Método:**

Estudo observacional, retrospectivo, caso-controle. Foram avaliados prontuários eletrônicos de pacientes consecutivos ≥ 60 anos, hospitalizados com pneumonia por COVID-19 entre 1º de março e 15 de julho de 2020. Comparações feitas pelos testes `t’ de *Student* ou qui-quadrado. Foi empregado modelo de risco proporcional de Cox para identificação de preditores de FAN. Considerou-se o valor de p < 0,05 como estatisticamente significativo.

**Resultados:**

Entre 667 pacientes internados por COVID-19, 201 (30,1%) foram incluídos. FAN foi documentada em 29 pacientes (14,4%) (grupo 1). Grupo 2 foi composto por 162 pacientes que não apresentaram FAN. Dez pacientes excluídos por estarem em FA na admissão hospitalar. Houve diferenças entre os grupos 1 e 2, respectivamente, no tempo de permanência em UTI (11,1±10,5 dias vs. 4,9±7,5 dias; p=0,004), necessidade de ventilação invasiva (82,9% e 32,7%; p<0,001) e mortalidade hospitalar (75,9% vs. 32,1%; p<0,001). No modelo de Cox, idade > 71 anos ( *hazard ratio* [HR]=6,8; p<0,001), leucometria ≤ 7.720 cels.μL^-1^ (HR=6,6; p<0,001), natremia ≤ 137 mEq.L^-1^ (HR=5,0; p=0,001), escore SAPS3 > 55 (HR=5,6; p=0,002) e desorientação (HR=2,5; p=0,04) foram preditores independentes de FAN.

**Conclusões:**

FAN é uma arritmia comum em idosos hospitalizados com pneumonia por COVID-19. Parâmetros clínicos e laboratoriais avaliados na admissão são preditores de FAN durante internação.

## Graphical abstract

**Figure:**
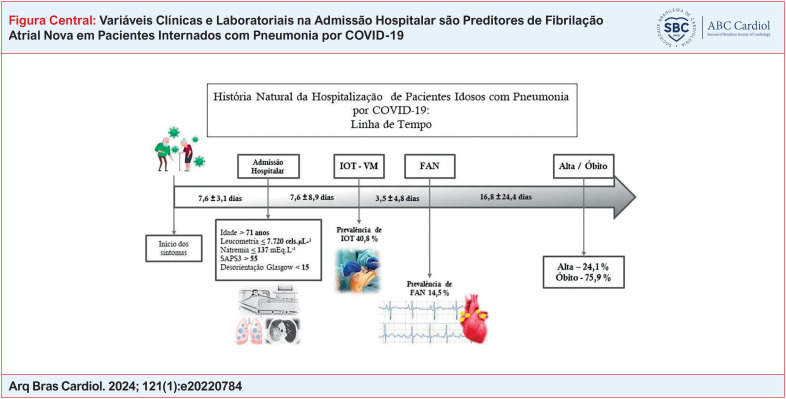
Cronograma de Hospitalização por Pneumonia COVID-19. A seta indica a direção dos acontecimentos após o início dos sintomas (da esquerda para a direita). Abaixo, os eventos mais relevantes após o início dos sintomas, com suas respectivas representações visuais. Na seta são apresentados os intervalos de tempo entre os eventos demarcados, como média±DP. Após o início dos sintomas, a internação hospitalar ocorreu, em média, em 7,3 ± 3,1 dias. Estão destacadas na caixa correspondente ao evento de Internação Hospitalar, as cinco características (preditores independentes e respetivos valores de corte óptimos) associadas ao desenvolvimento de Fibrilação Atrial Nova (FAN), ao longo do internamento. Observe os eventos subsequentes, intubação orotraqueal (IOT) e ventilação mecânica invasiva (VM), que precedem a ocorrência de FAN. Em média, a FAN ocorreu 3,5 ± 4,8 dias após IOT+VM. IOT: intubação orotraqueal; VM: ventilação mecânica invasiva; FAN: fibrilação atrial nova; SAPS3: Escore Simplificado de Fisiologia Aguda III.

## Introdução

A pandemia da doença pelo novo coronavírus que se iniciou no final de 2019 (COVID-19) com epicentro na cidade de Wuhan, na China, rapidamente se alastrou pelo mundo trazendo consequências catastróficas para saúde pública e economia global.^1^

Nomeado SARS-CoV-2, o novo coronavírus predispõe a uma pneumonia viral inicial, porém as principais consequências clínicas derivam da ampla reação inflamatória sistêmica.^2^ As principais manifestações clínicas são as pulmonares e cardiovasculares, e os indivíduos de pior prognóstico são aqueles mais idosos e previamente cardiopatas.^3 , 4^

A fibrilação atrial (FA) é uma arritmia cardíaca comum em pessoas acima de 55 anos^5^ e é frequentemente desencadeada em pacientes que apresentam contextos clínicos inflamatórios como nas miocardites.^6^

Na COVID-19, a ocorrência aguda de FA parece estar associada a um estado inflamatório sistêmico.^7^ Diversos estudos mostram a relação entre a COVID-19 e FA nova (FAN), sugerindo que a manifestação aguda da arritmia esteja associada a um prognóstico reservado.^8^ No momento da internação hospitalar, muitos pacientes se apresentam com quadros respiratórios graves, caracterizados por dispneia intensa e baixa saturação arterial de oxigênio, associados à pneumonia viral na tomografia computadorizada de tórax, reflexo de quadro inflamatório sistêmico avançado.

Portanto, neste cenário, é possível que informações clínicas, epidemiológicas e laboratoriais, avaliadas na admissão hospitalar, tenham valor prognóstico para o desenvolvimento da FAN, durante a internação.

O objetivo do presente trabalho foi investigar o valor preditivo de variáveis clínicas, epidemiológicas e laboratoriais obtidas na admissão hospitalar para o desenvolvimento de FAN em pacientes idosos internados com pneumonia por COVID-19.

## Métodos

### Delineamento

Trata-se de um estudo observacional, quantitativo, longitudinal, caso-controle, com análise retrospectiva de dados de prontuários eletrônicos coletados prospectivamente, no período entre os dias 01 de março a 15 de julho de 2020, de uma coorte de pacientes consecutivos internados no Hospital Universitário Pedro Ernesto (HUPE), da Universidade do Estado do Rio de Janeiro, com diagnóstico de COVID-19.

Este estudo foi aprovado no Comitê de Ética em Pesquisa sob o número CAAE 35192920.2.0000.528, e foi dispensado o uso de consentimento livre e esclarecido.

### Variáveis analisadas

As variáveis analisadas neste trabalho foram coletadas na admissão do paciente ao hospital.

A admissão hospitalar foi definida como primeira avaliação completa, nas primeiras 24 horas da internação, incluindo avaliação médica, laboratorial e de exames de imagens. A data da admissão hospitalar foi definida como a data de admissão do paciente na unidade COVID-19.

Os pacientes admitidos tiveram três origens principais: i – pacientes advindos do sistema de regulação municipal ou estadual com quadro suspeito ou confirmado de COVID-19; ii – pacientes advindos do próprio domicílio para triagem no hospital, e; iii – pacientes hospitalizados por outros motivos que desenvolveram a infecção durante a internação.

Os pacientes admitidos foram internados em unidades de terapia intensiva ou enfermaria, especialmente desenvolvidas para seu acolhimento.

#### Variáveis Demográficas

As variáveis demográficas analisadas foram idade e sexo.

#### Comorbidades Clínicas

As informações referentes às comorbidades clínicas foram extraídas por meio de busca de palavras-chave textuais e suas variações e abreviações, utilizando um algoritmo de busca ativa incluindo espaços, especialmente desenvolvido para este fim. Estes dados foram coletados e estão disponíveis no banco de dados *Red-cap* na Faculdade de Ciências Médicas da Universidade do Estado do Rio de Janeiro. Para assegurar a qualidade das informações, os dados foram triplamente checados por um especialista.

As comorbidades clínicas coletadas e analisadas foram: hipertensão arterial sistêmica (HAS), diabetes mellitus (DM), doença arterial coronariana (DAC), doença renal crônica (DRC), doença pulmonar obstrutiva crônica (DPOC), asma, obesidade e índice de massa corpórea (IMC), tabagismo, neoplasia, doença hepática crônica, doença autoimune, doença imunológica, doença hematológica crônica, doença neurológica crônica, insuficiência cardíaca prévia (IC). O número total de comorbidades também foi uma variável avaliada para cada paciente.

### Medicamentos

Alguns medicamentos de uso prévio que sugeriam se relacionar com mecanismos fisiopatológicos da COVID-19 foram analisados, como o inibidor da enzima conversora de angiotensina (IECA) ou bloqueador do receptor de angiotensina II (BRA),^9^ metformina,^10^ estatinas^11^ e anti-inflamatórios não esteroides (AINES).

### Sinais e sintomas na admissão hospitalar

Os sinais e sintomas foram coletados da anamnese médica na admissão do paciente. Estas variáveis foram: febre, tosse, disosmia, disgeusia, astenia, coriza, dispneia, desorientação, agitação ou sonolência, odinofagia, mialgia, diarreia, náuseas, vômitos, cefaleia, sincope, hipotensão e saturação de oxigênio (SpO2) abaixo de 94%.

A hipotensão era caracterizada como pressão arterial sistólica abaixo de 100 mmHg. Dispneia foi uma informação no prontuário de uma queixa subjetiva relatada pelo paciente ou pelos familiares. A variável desorientação foi definida usando a escala de Glasgow menor que 15, conforme descrito em prontuário.

### Parâmetros clínico-hospitalares

Foram também coletadas para análise as seguintes informações durante a internação: data de admissão em unidade de terapia intensiva (UTI), necessidade de ventilação mecânica invasiva, ocorrência de choque por qualquer causa e necessidade de terapia de substituição renal. Além disso, foram coletados os tempos, em dias, desde o início dos sintomas até a data de internação hospitalar, total de internação hospitalar, de internação em UTI, desde a internação até o início da ventilação mecânica, total de permanência em ventilação mecânica e o da internação até o primeiro episódio de FA.

Foram avaliados os escores de gravidade clínica SOFA ( *Sequential Organ Failure Assessment* )^12^ e SAPS3 ( *Simplilfied Acute Physiology Score 3* ),^13^ no momento da admissão hospitalar.

Utilizou-se a escala de Lawton para avaliação da fragilidade clínica do idoso, uma vez que esta avalia as atividades instrumentais da vida cotidiana.^14^

### Variáveis laboratoriais

Os exames laboratoriais avaliados na admissão hospitalar do paciente foram: Dímero-D, desidrogenase lática, hematócrito, hemoglobina, leucograma, contagem de linfócitos, contagem de plaquetas, transaminase oxalacética e pirúvica, ureia, creatinina, natremia, potássio, ferritina, troponina I, triglicerídeos, colesterol total e frações de alta e baixa densidade, fibrinogênio, relação normatizada internacional (RNI), precursor do peptídeo natriurético cerebral (NT-pró-BNP), bilirrubina total, glicemia, proteínas totais, albumina plasmática, creatinofosfoquinase, taxa de filtração glomerular pelo CKD-EPI.

#### Exames de Imagem

##### Tomografia Computadorizada de Tórax

Os exames de tomografia computadorizada (TC) de tórax foram realizados no dia da admissão hospitalar ou no dia seguinte e as imagens foram disponibilizadas para consulta online. Os aparelhos utilizados foram Brilliance 64 canais (Philips, Netherlands), SOMATOM Scope 16 canais (Siemens Healthcare GmbH, Germany) e Revolution ACT 16 canais (General Electrics, USA).

Alguns pacientes foram transferidos para o HUPE com laudo da TC de tórax realizada no hospital de origem. Nestes casos, a informação do laudo foi obtida do prontuário.

Os padrões pulmonares da TC de tórax utilizados para classificar os pacientes com quadro de pneumonia viral associada ao COVID-19 foram descritos por Mogami et al. e caracterizavam-se pelo padrão típico de opacidades em pontilhado hiperdenso tipo milharia, focal, em geral distribuído perifericamente nas regiões pulmonares, designados genericamente como “vidro fosco”.^15^

No presente trabalho, a gravidade da manifestação pulmonar, foi caracterizada pelo percentual da área do parênquima pulmonar acometido com padrão de “vidro fosco”. A reconstrução tomográfica bidimensional foi analisada desde o ápice até a base em cortes com espessuras que variaram de 3 a 5 mm.

O cálculo da área acometida total em vidro fosco foi feito pelo delineamento do percentual de acometimento de cada corte tomográfico, somado ao longo de todos os cortes. Esta soma posteriormente foi dividida pelo número de cortes traçando assim o percentual médio do acometimento pulmonar.

Para as análises foi feita a dicotomização definida arbitrariamente no limite de 50%, sendo ≥ 50% e < 50%.

##### Ecocardiograma

Avaliação das dimensões das cavidades cardíacas e da função ventricular direita e esquerda foi realizada utilizando equipamento de ultrassom portátil InnoSight™ (Phillips, Netherlands) pelo protocolo “ *point of care ultrasound* ” (POCUS).

As avaliações ecocardiográficas realizadas à beira de leito pelo POCUS foram feitas pela avaliação subjetiva da dimensão cavitária, contratilidade segmentar e da função sistólica global do ventrículo esquerdo (VE).

Definiu-se presença de disfunção ventricular esquerda (VE) quando o ecocardiograma transtorácico à beira do leito pelo POCUS demonstrava alterações características (hipocontratilidade global ou segmentar das paredes do VE) ou quando o NT-pro-BNP estava elevado.

Como em muitos pacientes não houve registro quantitativo da fração de ejeção, optou-se por utilizar a análise qualitativa disponível da função ventricular (disfunção ventricular).

### Extração de Dados

Os dados foram extraídos do sistema padronizado de prontuário eletrônico (MVPEP) e o critério inicial utilizado para identificação do paciente era estar internado no HUPE em unidade COVID-19 e com o diagnóstico confirmado pelo exame RT-PCR.

A extração dos dados foi feita a partir do dia 01 de março de 2020 até o dia 15 de julho de 2020.

Os dados coletados inicialmente foram o número do prontuário, nome completo, a primeira unidade de internação, número do atendimento (código da autorização de internação hospitalar – AIH), idade, sexo, etnia, data de internação, data da alta/óbito, movimentação entre unidades durante internação com as respectivas datas de admissão em cada unidade, data da realização do RT-PCR e seu resultado.

Para seleção dos pacientes com FAN foi necessário o registro da ocorrência de ritmo sinusal ou ritmo regular na admissão e ritmo de fibrilação atrial, durante a internação.

A ocorrência de FAN foi identificada considerando os seguintes pontos de informação dos prontuários: i – registro nas evoluções médicas no prontuário eletrônico com avaliação seriada do ritmo cardíaco; ii – registros de laudos descritivos de eletrocardiograma (ECG) no prontuário eletrônico e; iii – visualização de traçados de ECG.

No caso do registro do ECG impresso não estar disponível no prontuário, considerou-se a ocorrência de FAN, quando esta era relatada na data do evento e em evoluções médicas subsequentes.

### Critérios de Inclusão e Exclusão

Todos os pacientes deste estudo apresentaram somente uma internação hospitalar no período avaliado.

Uma comissão médica revisora composta por especialistas do Hospital Universitário Pedro Ernesto foi instituída para avaliar todos os prontuários eletrônicos dos pacientes internados com diagnóstico presumido de COVID-19 até 15 de julho de 2020. A comissão era composta por um geriatra, um reumatologista, um pneumologista e um infectologista.

Os critérios de inclusão para o presente estudo foram:

- Idade de 60 anos ou mais.
- Diagnóstico molecular confirmado de COVID-19 pelo RT-PCR.
- Diagnóstico por imagem de pneumonia viral por COVID-19.
- Nos pacientes que tiveram FAN, era necessária a documentação de pelo menos um episódio de FA durante o período de internação.

Foram excluídos os pacientes:

- Com idade inferior a 60 anos.
- Sem diagnóstico molecular confirmado de COVID-19.
- TC de tórax não disponível.
- Documentação de ritmo de FA à admissão hospitalar.

### Desfecho Clínico Fibrilação Atrial Nova (FAN)

Para fins de avaliação prognóstica, a FAN foi tratada como desfecho primário.

### Análises Estatísticas

#### Comparação de variáveis

As variáveis contínuas foram apresentadas como média ± desvio padrão (DP) e as variáveis categóricas, como porcentagem ou razão. Os pacientes que desenvolveram FAN foram designados como grupo 1 e os que não desenvolveram a arritmia formaram o grupo 2. A distribuição de probabilidade das variáveis foi avaliada pelo teste de assimetria de Pearson. As variáveis que apresentavam valor absoluto maior que 3 foram consideradas não-normais, sendo, portanto, logaritmizadas para comparação estatística, a fim de normalizar as distribuições de probabilidade. As variáveis numéricas normalmente distribuídas foram comparadas entre os grupos por meio do teste t de Student não emparelhado. A comparação das variâncias para aplicação adequada do teste t foi realizada pelo teste de Levene ou da variável F de Snedecor, quando necessário. As variáveis categóricas foram comparadas pelo teste qui-quadrado ou pelo teste exato de Fisher. Foram calculados risco relativo (RR) ou *odds ratio* (OR) e respectivos intervalos de confiança (IC) de 95%, quando apropriado.

Os aplicativos utilizados para as análises estatísticas foram Medcalc v. 10.3.2 (MedCalc Software Ltd, Bélgica) e Microsoft Excel 2021 e 365 (Microsoft Corporation, Redmond, Washington, EUA) com suplemento XRealStats (Real Stattistcs Using Excel. https://www.real-statistics.com/, último acesso em 06/10/2022).

#### Modelagem estatística

Utilizou-se a análise da curva *ROC* para a dicotomização das variáveis numéricas e definir o valor de corte ótimo. Para investigação do valor prognóstico das variáveis selecionadas com a ocorrência de FAN, foi usado o modelo de risco proporcional de Cox e a regra de uma variável dependente no modelo para cada cinco desfechos FAN. Após análise de Cox para o desfecho FAN foi feita a curva de Kaplan-Meier das respectivas variáveis significativas.

Foi de interesse avaliar as variáveis obtidas na admissão como indicadores da evolução intra-hospitalar até o desenvolvimento da FAN. Utilizaram-se, assim, as análises uni e multivariada, considerando tempo de evolução desde a admissão hospitalar até a ocorrência da FAN. As variáveis significativas no modelo univariado foram admitidas em um modelo multivariado para identificação de preditores independentes do desfecho FAN. As variáveis preditoras independentes foram admitidas para o desenvolvimento de um sistema de pontuação, a fim de avaliar o risco de FAN. Para este fim, os coeficientes beta das variáveis significativas da equação de regressão foram truncados ao inteiro mais próximo. Assim, no sistema de pontuação, cada variável independente passou a ter dois valores atribuíveis: zero ou o valor obtido pela aproximação de seu coeficiente beta. Considerando a composição do sistema de pontuação como a soma dos valores atribuídos a cada variável, o valor mínimo possível era zero e máximo, a soma dos valores atribuídos de cada variável.

Foi realizada análise *post-hoc* para calcular a potência estatística (1 - β) atingida no presente estudo, utilizando o teste do qui-quadrado para comparações entre grupos 1 e 2. Foram utilizadas as razões dos quantitativos de cada grupo para calcular o valor da potência atingida. O nível de erro alfa foi definido em 0,05 para todos os testes estatísticos.

## Resultados

**Figura 1:**
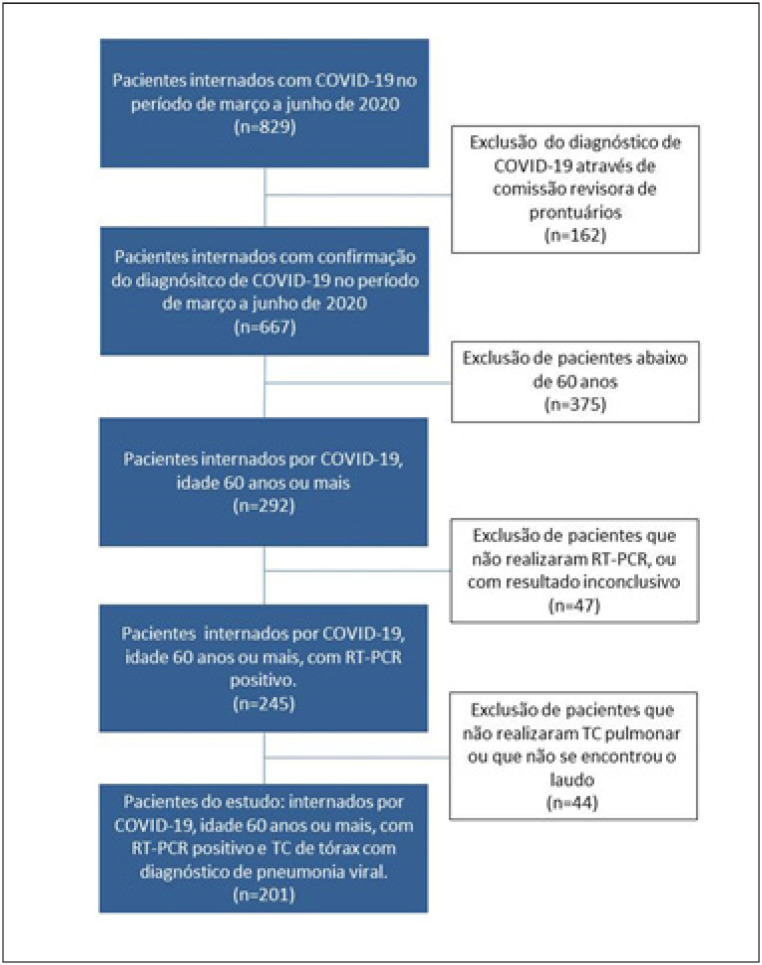
Fluxograma para inclusão dos pacientes.

No período de 1º de março a 15 de julho de 2020, 667 pacientes foram internados por COVID-19, após avaliação da comissão revisora. Destes, 201 (30.1%) preencheram os critérios de inclusão para este estudo ( figura 1 ). A FAN foi documentada em 39 pacientes (19,4%), sendo 10 indivíduos excluídos da análise por se apresentarem em FA no momento da admissão. Assim, 29 pacientes (14,4%) foram classificados como grupo 1 e 162 pacientes, que não desenvolveram FAN, grupo 2. Um dos pacientes do grupo 1 apresentou apenas registro ao ECG de ritmo de flutter atrial e foi incluído na análise. O tempo médio de ocorrência de FAN após a admissão hospitalar foi de 13,7 ± 14,0 dias e o tempo total de internação hospitalar nos grupos 1 e 2 foi, respectivamente, de 30,5 ± 27,6 dias e 16,2 ± 12,4 dias. No grupo 1, 89,7% dos pacientes estavam em ritmo sinusal ao final da internação.

Assim, a FAN se associou a um contexto de maior gravidade e um resumo da evolução da história natural destes pacientes é apresentado na linha do tempo (Figura Central).

Em relação aos dados clínicos e demográficos, os indivíduos do grupo 1 tinham idade mais avançada, apresentavam maior prevalência de doença neurológica crônica e maior número de comorbidades, em comparação ao grupo 2 ( Tabela 1 ).

**Tabela 1:** Características epidemiológicas e clínicas da população à ocasião da internação

Variáveis	FAN (n=29)	Não-FAN (n=162)	Valor de p	RR para FAN
Idade (anos) *	73,9 ± 8,5	69,8 ± 7,4	0,008 †	
Sexo feminino (n (%))	8 (27,6)	69 (42,6)	0,189 ‡	0,56 IC 95% [0,26 – 1,19]
Hipertensão arterial sistêmica (n (%))	24 (82,8)	118 (72,8)	0,326 ‡	1,56 IC 95% [0,63 – 3,86]
Diabetes mellitus (n (%))	11 (37,9)	73 (45,1)	0,574 ‡	0,75 IC 95% [0,38 – 1,50]
Doença arterial coronária (n (%))	8 (28,6)	28 (17,6)	0,248 ‡	1,55 IC 95% [0,74 – 3,23]
Doença renal crônica (n (%))	5 (17,2)	24 (14,8)	0,738 ‡	1,09 IC 95% [0,45 – 2,62]
Doença pulmonar obstrutiva crônica (n (%))	4 (13,8)	15 (9,3)	0,647 ‡	1,38 IC 95% [0,54 – 3,53]
Asma (n (%))	0 (0,0)	2 (1,2)	0,924 ‡	N/A
Obesidade (n (%))	8 (30,8)	35 (26,7)	0,755 ‡	1,13 IC 95% [0,53 – 2,40]
IMC (kg/m^2^) *	28,1 ± 14,2	28,4 ± 14,7	0,674 §	
Tabagismo (n (%))	8 (29,6)	48 (31,4)	0,868 ‡	0,88 IC 95% [0,41 – 1,90]
Neoplasia (n (%))	2 (6,9)	27 (16,9)	0,264 ‡	0,38 IC 95% [0,10 – 1,52]
Doença hepática crônica (n (%))	3 (10,3)	10 (6,3)	0,640 ‡	1,45 IC 95% [0,51 – 4,16]
Doenças autoimunes (n (%))	2 (7,1)	4 (2,5)	0,484 ‡	2,32 IC 95% [0,71 – 7,60]
Doenças imunológicas (n (%))	3 (10,3)	12 (7,5)	0,780 ‡	1,25 IC 95% [0,43 – 3,67]
Doença hematológica crônica (n (%))	1 (3,4)	5 (3,1)	0,635 ‡	1,10 IC 95% [0,18 – 6,81]
Doença neurológica crônica (n (%))	9 (31,0)	16 (9,9)	0,005 ‡	2,87 IC 95% [1,48 – 5,59]
Insuficiência cardíaca prévia (n (%))	8 (27,6)	21 (12,9)	0,067 ‡	1,82 IC 95% [0,89 – 3,70]
Outras comorbidades (n (%)) †	18 (64,3)	75 (48,7)	0,145 ‡	1,57 IC 95% [0,77 – 3,21]
Número de comorbidades antes da internação *	4,1 ± 1,4	3,4 ± 1,7	0,005 ¶	
IECA/BRA (n (%))	18 (62,1)	94 (58,0)	0,608 ‡	1,08 IC 95% [0,54 – 2,15]
Metformina (n (%))	5 (17,2)	31 (19,1)	0,994 ‡	0,87 IC 95% [0,36 – 2,13]
Sinvastatina (n (%))	11 (37,9)	58 (36,0)	0,709 ‡	1,01 IC 95% [0,51 – 2,02]
AINES (n (%))	2 (8,0)	5 (3,3)	0,533 ‡	1,83 IC 95% [0,53 – 6,26]
Número de medicamentos antes da internação *	3,6 ± 2,6	3,1 ± 3,0	0,186 ¶	
Escada de Lawton antes da internação *	15,8 ± 9,0	18,0 ± 9,3	0,099 ¶	
SOFA na admissão*	5,6 ± 4,2	4,4 ± 3,9	0,013 ¶	
SAPS3 na admissão*	60,6 ± 28,1	54,9 ± 29,1	0,037 ¶	
Acometimento pulmonar >=50% na admissão (n (%))	16 (55,2)	78 (48,1)	0,483 ‡	1,18 IC 95% [0,06 – 2,32] †

IECA: inibidor da enzima conversora de angiotensina; BRA: bloqueador do receptor da angiotensina II; AINES: anti-inflamatórios não-esteroidais; IMC: índice de massa corpórea ; FAN: fibrilação atrial nova; RR: risco relativo.

* média ± DP.

† Em outras comorbidades incluíram-se: alcoolismo, ex-tabagismo, mal de Alzheimer, artrite reumatoide e vasculite, doença cerebrovascular, demência, depressão, dislipidemia, gota, hipotireoidismo, osteoporose, doença arterial periférica, aneurisma de aorta, história de trombose venosa profunda.

‡ Comparação pelo teste do qui-quadrado.

§ Comparação pelo teste t de Student não emparelhado.

¶ Comparação pelo teste Mann-Whitney.

Em relação aos sintomas registrados na admissão hospitalar, no grupo 1 foram registradas maiores frequências de mialgia e desorientação. Os demais sintomas comuns da COVID-19 não apresentaram diferença entre grupos ( Tabela 2 ).

**Tabela 2:** Características dos sintomas da população antes da internação

Variáveis	FAN (n=29)	Não-FAN (n=162)	Valor de p	RR para FAN
Febre (n (%))	23 (79,3)	108 (66,7)	0,243	1,69 IC 95% [0,73 – 3,94]
Tosse (n (%))	15 (51,7)	111 (68,5)	0,115	0,53 IC 95% [0,27 – 1,03]
Disosmia (n (%))	4 (13,8)	12 (7,4)	0,436	1,75 IC 95% [0,70 – 4,40]
Disgeusia (n (%))	1 (3,4)	6 (3,7)	0,778	0,82 IC 95% [0,13 – 5,18]
Astenia (n (%))	11 (37,9)	70 (43,2)	0,689	0,80 IC 95% [0,40 – 1,60]
Mialgia (n (%))	13 (44,8)	37 (22,8)	0,025	2,20 IC 95% [1,14 – 4,25]
Coriza (n (%))	4 (13,8)	15 (9,3)	0,679	1,45 IC 95% [0,56 – 3,72]
Dispneia (n (%))	28 (96,6)	141 (87,0)	0,227	3,46 IC 95% [0,50 – 24,19]
Saturação de oxigênio < 94% (n (%))	27 (93,1)	147 (90,7)	0,836	1,25 IC 95% [0,33 – 4,82]
Desorientação (n (%))	13 (44,8)	35 (21,6)	0,016	2,28 IC 95% [1,18 – 4,38]
Agitação / Sonolência pré-internação (n (%))	9 (31,0)	42 (25,9)	0,626	1,17 IC 95% [0,57 – 2,39]
Odinofagia (n (%))	1 (3,4)	9 (5,6)	0,981	0,64 IC 95% [0,10 – 4,26]
Diarreia (n (%))	1 (3,4)	30 (18,5)	0,081	0,18 IC 95% [0,03 – 1,26]
Náuseas (n (%))	1 (3,4)	13 (8,0)	0,628	0,45 IC 95% [0,07 – 3,08]
Vômitos (n (%))	1 (3,4)	13 (8,0)	0,628	0,45 IC 95% [0,07 – 3,08]
Cefaleia (n (%))	2 (6,9)	17 (10,5)	0,795	0,67 IC 95% [0,17 – 2,60]
Síncope (n (%))	2 (6,9)	6 (3,7)	0,779	1,69 IC 95% [0,48 – 5,88]
Hipotensão (n (%))	1 (3,4)	13 (8,0)	0,623	0,45 IC 95% [0,07 – 3,06]

FAN: fibrilação atrial nova; RR: risco relativo.

Quanto aos indicadores de gravidade avaliados na admissão hospitalar, os pacientes do grupo 1 apresentaram, em relação ao grupo 2, escores significativamente mais elevados de SOFA e SAPS3 ( Tabela 1 ). Não houve diferenças entre os grupos na distribuição do acometimento pulmonar acima de 50% na admissão ( Tabela 1 ).

Adicionalmente, em relação à evolução hospitalar dos pacientes, como demonstrado na tabela 3 , os pacientes do grupo 1 apresentaram maior necessidade de internação em UTI e de uso de ventilação mecânica invasiva, bem como maior tempo de internação em UTI e maior tempo total de ventilação mecânica invasiva. Não houve diferenças na necessidade de terapia de substituição renal, no acometimento pulmonar acima de 50% e no tempo de início dos sintomas até a admissão hospitalar. O uso de medicações como noradrenalina, glicocorticoide, cloroquina e hidroxicloroquina foi significativamente maior no grupo 1. Não houve significância estatística na associação do uso de outros medicamentos avaliados e ocorrência de FAN ( Tabela 3 ).

**Tabela 3:** Parâmetros clínicos e hospitalares, e terapias farmacológicas utilizadas durante internação hospitalar

Parâmetros clínicos e hospitalares	FAN (n=29)	Não-FAN (n=162)	Valor de p	OR para FAN
Admissão na UTI (n (%))	28 (96,6)	80 (49,4)	<0,001	27,01 IC 95% [3,59 – 203,28]
Ventilação mecânica (n (%))	24 (82,8)	53 (32,7)	<0,001	90,2 IC 95% [3,26 – 24,96]
Terapia de substituição renal (n (%))	10 (34,5)	32 (19,8)	0,116	1,90 IC 95% [0,81 – 4,48]
Tempo de início de sintomas até a data de internação hospitalar (dias) *	7,6 ± 4,3	8,5 ± 5,1	0,380	
Tempo total de internação hospitalar (dias) *	30,5 ± 27,6	16,2 ± 12,4	0,010	
Tempo de internação em UTI (dias) *	11,1 ± 10,5	4,9 ± 7,5	0,004	
Tempo da internação hospitalar até início da ventilação mecânica (dias) *	7,6 ± 9,2	4,2 ± 5,1	0,101	
Tempo em ventilação mecânica (dias) *	12,9 ± 14,0	9,5 ± 5,9	0,008	
Tempo da internação até o primeiro episódio de FA (dias) *	13,7 ± 14,0	-	-	
Noradrenalina (n (%))	27 (93,1)	60 (37,0)	<0,001	22,95 IC 95% [5,27 – 99,95]
Cloroquina / Hidroxicloroquina (n (%))	13 (44,8)	42 (25,9)	0,042	2,32 IC 95% [1,03 – 5,23]
Uso de glicocorticoide (n (%))	23 (79,3)	66 (40,7)	<0,001	5,58 IC 95% [2,15 – 14,44]
Heparina (não fracionada ou de baixo peso molecular) (n (%))	26 (89,7)	139 (86,5)	0,628	1,37 IC 95% [0,38 – 4,92]
Ivermectina (n (%))	11 (37,9)	44 (27,2)	0,241	1,64 IC 95% [0,72 – 3,74]

UTI: Unidade de Terapia Intensiva; FAN: fibrilação atrial nova.

* média ± DP.

Não houve diferenças na ocorrência de complicações intra-hospitalares analisadas entre os grupos 1 e 2, exceto na taxa de infecção nosocomial não-pulmonar e no choque onde foram significativamente maiores no grupo 1 (Tabela 4). A taxa de mortalidade hospitalar nos pacientes do grupo 1 e grupo 2 foram, respectivamente, de 75,9% vs. 32,1% com RR para óbito de 2,36 ( Tabela 4 ).

**Tabela 4:** Análise das razões de chance das complicações clínicas intra-hospitalares (acima), e análise do risco relativo de óbito (abaixo) nos grupos FAN e Não-FAN

Variáveis	FAN (n=29)	Não-FAN (n=162)	Valor de p	OR para FAN
Coagulação intravascular disseminada (n (%))	0 (0)	1 (0,6)	0,327	
Trombose venosa profunda (n (%))	1 (3,6)	5 (3,1)	0,653	
Tromboembolismo pulmonar (n (%))	0 (0)	3 (1,9)	0,654	
Infecção pulmonar hospitalar (n (%))	15 (51,7)	62 (39,2)	0,259	
Infecção hospitalar de qualquer natureza não-pulmonar (n (%))	13 (44,8)	24 (14,8)	<0,001	4,67 IC 95% [1,79 – 6,40]
Delirium (n (%))	6 (20,7)	42 (25,9)	0,579	
Disfunção ventricular esquerda (n (%))	16 (55,2)	90 (55,6)	0,842	0,83 IC 95% [0,37 – 1,84]
Choque (n (%))	18 (62,1)	44 (27,2)	<0,001	4,39 IC 95% [1,92 – 10,03]
Variáveis	FAN (n=29)	Não-FAN (n=162)	Valor de p	Risco Relativo
Óbito (n (%))	22 (75,9)	52 (32,1)	<0,001	2,36 IC 95% [1,74 – 3,20]

OR: odds ratio; FAN: fibrilação atrial nova.

As variáveis laboratoriais intra-hospitalares foram comparadas entre os grupos e são demonstradas na tabela 5 . O grupo 1 apresentou menores valores no leucograma, contagem de plaquetas, TGO, sódio sérico e proteínas séricas totais. Além disso, o percentual de pacientes com troponina I com valor > 0,2 ng.mL^-1^ foi maior no grupo 1.

**Tabela 5:** Exames laboratoriais na admissão hospitalar

Variáveis †	FAN (n=29)	Não-FAN (n=162)	Valor de p	RR para FAN
Dímero-D (ng.mL^-1^) † *	6181,9 ± 15112,2	5120,4 ± 11443,3	0,723	
LDH (U.L^-1^) †	383,0 ± 194,4	448,8 ± 251,7	0,199	
HTO (%) †	36,3 ± 5,7	35,8 ± 6,9	0,736	
HGB (g.dL^-1^) †	12,1 ± 1,9	11,8 ± 2,5	0,469	
LEU (cels.μL^-1^) †	6726,2 ± 2894,0	9482,4 ± 10398,1	0,005	2,63 IC 95% [1,18–5,86]
LINF (%) †	13,7 ± 8,4	15,4 ± 11,0	0,418	
PLA (cels.μL^-1^) †	188,1 ± 96,9	252,4 ± 116,7	0,006	4,15 IC 95% [1,94–8,88]
TGO (U.L^-1^) †	38,4 ± 19,6	59,0 ± 111,8	0,036	2,45 IC 95% [1,20–5,02]
TGP (U.L^-1^) † *	30,3 ± 15,2	43,4 ± 56,8	0,224	
GGT (U.L^-1^) † *	107,4 ± 86,6	139,2 ± 168,8	0,193	
URE (mg.dL^-1^) †	60,5 ± 48,0	60,8 ± 49,3	0,979	
FER (pmol.L^-1^) † *	1611,2 ± 1225,8	2005,7 ± 3087,3	0,328	
CRE (mg.dL^-1^) †	1,4 ± 1,0	1,8 ± 2,0	0,162	
Tn I (>0,2 ng.mL^-1^)	30,2 %	12,2 %	0,038	2,46 IC 95% [1,07–5,69]
FIB (mg.dL^-1^) †	537,8 ± 270,8	480,5 ± 200,3	0,264	
RNI (AU) †	1,3 ± 0,2	1,2 ± 0,2	0,111	
NT-pro-BNP (pg.mL^-1^) †	3884,0 ± 8619,2	5172,2 ± 8659,4	0,602	
PTN C R (mg.L^-1^) †	85,1 ± 88,6	58,2 ± 52,6	0,128	
BAST (contagem absoluta) †	92,0 ± 254,1	181,2 ± 657,3	0,205	
PTT (segundos) †	31,3 ± 9,9	29,3 ± 5,9	0,300	
K (mEq.L^-1^) †	4,5 ± 0,9	4,4 ± 0,8	0,538	
NA (mEq.L^-1^) †	136,7 ± 5,9	139,8 ± 6,8	0,019	2,51 IC 95% [1,26–5,01]
BIL (mg.dL^-1^) †	0,7 ± 0,4	0,7 ± 0,8	0,560	
GLI (mg.dL^-1^) †	171,5 ± 111,8	156,5 ± 120,1	0,576	
PTN (g.dL^-1^) †	5,9 ± 0,7	6,4 ± 1,4	0,042	2,75 IC 95% [1,33–5,68]
ALB (g.dL^-1^) †	2,8 ± 0,6	3,1 ± 0,6	0,089	
CPK (U.L^-1^) †	179,5 ± 243,5	223,3 ± 528,8	0,526	
TRI (mg.dL^-1^) † *	140,2 ± 51,0	156,4 ± 76,0	0,751	
COL (mg.dL^-1^) †	150,2 ± 38,3	144,7 ± 46,3	0,578	
HDL (mg.dL^-1^) † *	32,0 ± 6,4	34,9 ± 13,7	0,947	
LDL (mg.dL^-1^) †	93,2 ± 32,2	86,5 ± 41,2	0,459	
TGF CKD-EP (ml/min) †	79,0 ± 20,8	82,8 ± 25,7	0,460	

ALB: albumina plasmática; BAS: contagem absoluta de bastões; BIL: bilirrubina total; CRE: creatinina; COL: colesterol total; CPK: creatinofosfoquinase; FER: ferritina; FIB: fibrinogênio; GLI: glicemia; GGT: gama-glutamil transferase; HGB: hemoglobina; HDL: High Density Lipoprotein; HTO: hematócrito; K: potássio; LDH: desidrogenase lática; LDL: Low Density Lipoprotein; LEU: leucograma; LINF: contagem relativa de linfócitos; NA: Natremia; NT-pró-BNP: precursor do peptídeo natriurético cerebral; PLA: contagem de plaquetas; PTN: proteínas totais; PtnCR: proteína C reativa; PTT: tempo de tromboplastina parcial; RNI: relação normatizada internacional; TGF CKD-EPI: taxa de filtração glomerular pelo CKD-EPI; TGO: transaminase oxalacética; TGP: transaminase pirúvica; TnI: troponina I; TRI: triglicerídeos; URE: ureia.

* teste comparativo de Mann-Whitney.

† média ± DP.

### Análise e modelagem prognóstica para FAN

A análise da curva *ROC* definiu os valores de corte das variáveis que foram: idade > 71 anos (sensibilidade 69%, especificidade 62,1%), número de comorbidades > 4 (sensibilidade 48,3%, especificidade 77,0%), SAPS 3 > 55 (sensibilidade 75,0%, especificidade 77,7%), leucometria ≤ 7.720 cels.μL^-1^ (sensibilidade 75,9%, especificidade 49,7%), plaquetas ≤ 196.000 cels.μL^-1^ (sensibilidade 72,4%, especificidade 67,7%), TGO ≤ 37 U.L^-1^ (sensibilidade 64,3%, especificidade 62,1%), natremia ≤ 137 mEq.L^-1^ (sensibilidade 62,1%, especificidade 64,4%), proteínas totais ≤ 6 g.dL^-1^ (sensibilidade 66,7%, especificidade 64,5%). As variáveis assim dicotomizadas, bem como as variáveis categóricas significativas, foram analisadas por meio do modelo de risco proporcional de Cox univariada, com resultados apresentados na Tabela 6 .

**Tabela 6:** Análise uni e multivariada, de preditores da ocorrência de FAN por regressão proporcional de Cox (acima) e valor ótimo de corte do escore de avaliação de risco para ocorrência de FAN (abaixo) *

	Análise Univariada			Análise Multivariada			
	β	HR IC 95%	Valor de p	β	HR IC 95%	Valor de p	Escore
Idade > 71 anos	1,200	3,3 [1,5 – 7,3]	0,003	1,913	6,8 [2,5 – 18,3]	<0,001	1
Número de comorbidades > 4	0,840	2,3 [1,1 – 4,8]	0,024				
Doença neurológica	1,150	3,2 [1,4 – 7,0]	0,005				
Desorientação	1,179	3,3 [1,5 – 6,9]	0,002	0,919	2,5 [1,05 – 6,0]	0,04	1
Mialgia	1,007	2,7 [1,3 – 5,7]	0,007				
Infecção nosocomial	1,025	2,8 [1,3 – 5,8]	0,007				
Choque	1,178	3,2 [1,5 – 7,0]	0,003				
Ventilação mecânica	1,902	6,7 [2,6 – 17,5]	<0,001				
Internação em UTI	2,891	18,0 [8,5 – 131,5]	0,005				
Troponina > 0,2 ng.mL^-1^	0,651	1,9 [0,8 – 4,8]	0,170				
Uso de noradrenalina	2,622	13,8 [3,3 – 57,8]	<0,001				
SAPS3 > 55	1,173	3,2 [1,3 – 8,1]	0,013	1,723	5,6 [1,9 – 16,6]	0,002	1
Leucometria ≤ 7.720 cels.μL^-1^	1,170	3,2 [1,4 – 7,6]	0,008	1,887	6,6 [2,2 – 19,9]	<0,001	1
Plaquetas ≤ 196.000 cels.μL^-1^	1,503	4,5 [2,0 – 10,1]	<0,001				
TGO ≤ 37 U.L^-1^	0,986	2,7 [1,2 – 5,8]	0,014				
Natremia ≤ 137 mEq.L^-1^	0,836	2,3 [1,1 – 4,9]	0,030	1,611	5,0 [1,9 – 13,1]	0,001	1
Proteínas totais ≤ 6 g.dL^-1^	1,247	3,5 [1,5 – 8,0]	0,004				
Valor de corte ótimo	Especificidade(%) §	Sensibilidade(%) §	AUC §	Valor de p †	HR ‡ §		
> 2	65,2 [57,3 – 72,5]	82,8 [64,2 – 94,1]	0,815 [0,717 – 0,913]	<0,001	7,6 [2,9 – 19,8]		

HR: hazard ratio; IC: intervalo de confiança; FAN: fibrilação atrial nova; TGO: transaminase oxalacética.

* N do grupo 1 = 29; N do grupo 2 = 162.

† Valor de p do ajuste da função de Cox < 0,001; χ^2^ 15,0; r^2^=0,927.

‡ N=135;

§ [IC 95%]

**Figura 2:**
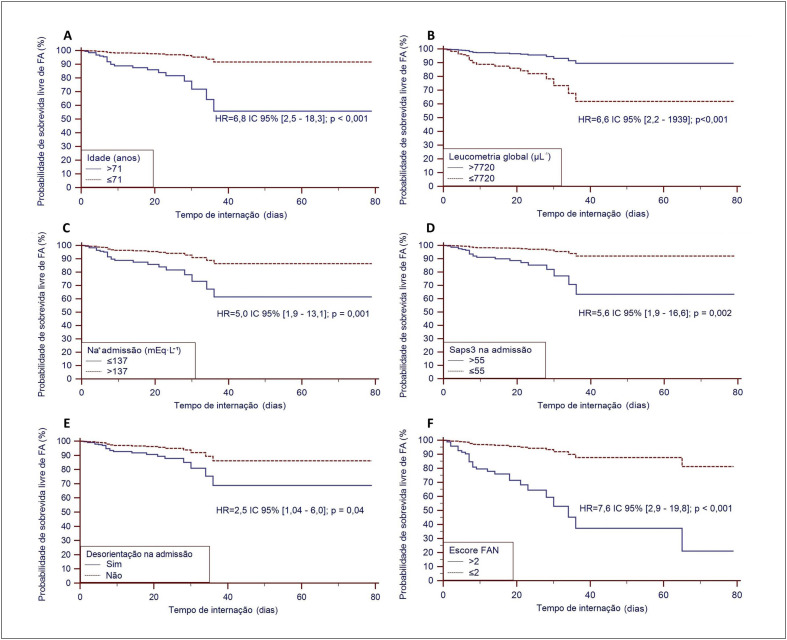
Curvas de Kaplan-Meier da probabilidade de sobrevida livre de FAN das variáveis colhidas na admissão hospitalar preditoras independentes de FAN (analisadas pelo modelo multivariado de Cox) e a curva Kaplan-Meier do escore FAN: A - Idade; B - Leucometria global; C - Sódio sérico; D - Escore SAPS3; E - Desorientação; F - Escore FAN. FAN: fibrilação atrial nova, SAPS3: Simplified Acute Physiology Score III.

Na análise multivariada do modelo de risco proporcional de Cox, empregando as variáveis com valor de p significativo, demonstrou que idade > 71 anos, leucometria ≤ 7.720 cels.μL^-1^, natremia ≤ 137 mEq.L^-1^, SAPS 3 > 55 e desorientação foram preditores independentes para ocorrência de FAN durante internação ( Tabela 6 , Figura 2 ). A partir do resultado do modelo de Cox multivariado, foi desenvolvido um sistema de pontuação com o objetivo de avaliar o risco de ocorrência de FAN nestes pacientes. Assim, de acordo com valores de beta das variáveis, foram designadas pontuações de 1 para cada uma das variáveis quando na faixa de gravidade, ou zero quando fora desta faixa. Este escore resultou em 6 pontuações possíveis: 0 a 5 (tabela 6). A distribuição dos pacientes de acordo com a pontuação foi: 0 pontos = 8,9%; 1 ponto = 26,7%; 2 pontos = 30,4%; 3 pontos = 25,2%; 4 pontos = 5,2%; 5 pontos = 3,7%. Aplicando análise da curva ROC à distribuição da pontuação do escore, o ponto de corte ótimo foi > 2, com elevadas especificidade e sensibilidade (Tabela 6).Utilizando o modelo de risco proporcional de Cox, o HR do valor de corte > 2 para FAN foi de 7,6 ( Tabela 6 e Figura 2 ).

### Análise Post-Hoc da potência estatística da amostra

A análise da amostragem foi realizada *post-hoc* em relação às distribuições das variáveis preditoras independentes entre os grupos 1 e 2, considerando erro alfa de 0,05. A potência estatística (1 - β) atingida para os desfechos FAN, da variável idade > 71 anos foi 89%, leucometria ≤ 7.720 cels.μL^-1^ foi 73%, natremia ≤ 137 mEq.L^-1^ 76% e desorientação 73% e SAPS3 > 55 de 92%. Quanto ao escore para FAN, considerando a distribuição dos valores > 2 nos grupos 1 e 2 de, respectivamente, 82,8% e 34,6%, a análise *post-hoc* revelou a potência estatística > 99%.

## Discussão

No presente estudo a FAN se mostrou um evento frequente em pacientes internados com pneumonia por COVID-19, sendo observada em 14,4% dos pacientes em ritmo sinusal na admissão. Em 2021, uma pesquisa mundial mostrou que a ocorrência de FA e flutter atrial na população geral com COVID-19 foi de 9,0% sendo mais comum na Europa (21,9%).^16^ Um estudo brasileiro mostrou que a presença de arritmias em 241 pacientes consecutivos internados com COVID-19 foi de 8,7%, sendo 76,2% de arritmias atriais.^17^ Adicionalmente, em nosso estudo, 26 dos 29 pacientes que tiveram FAN (89,7 %) estavam em ritmo sinusal ou regular ao final da internação, indicando que a FAN foi um fenômeno clínico transitório na maioria dos pacientes. Além disso, a FAN se associou a um contexto de maior gravidade, quando avaliado por índices clínicos e laboratoriais, desde a admissão hospitalar (Figura Central).

De fato, Sans et al. evidenciaram que a FAN foi associada a um contexto de maior gravidade e prognóstico reservado em uma coorte de 160 pacientes consecutivos internados por COVID-19.^18^ Entretanto, estes autores acrescentaram os pacientes que apresentavam FA prévia ao grupo que não desenvolveu FAN, e compararam com o grupo de desenvolveu FAN. Em nosso trabalho, optamos por uniformizar os grupos, excluindo os pacientes com FA prévia da análise. Foram constituídos, assim, dois grupos homogêneos no contexto da arritmia atrial.

Em relação às variáveis demográficas, comorbidades prévias e sintomas registrados no momento da admissão, a idade dos pacientes, a doença neurológica crônica, número total de comorbidades, desorientação e mialgia, tiveram impacto significativo na análise univariada para ocorrência de FAN durante internação ( Tabela 2 ).

Os pacientes que apresentaram FAN tinham características de maior gravidade na admissão hospitalar, como avaliados nos escores SOFA e SAPS3, quando comparados aos pacientes que não apresentaram FAN ( Tabela 1 ). Estas condições se refletiram em admissão em UTI com maior frequência e maior necessidade de ventilação mecânica invasiva ( Tabela 3 ), ainda que as alterações pulmonares na TC de tórax de admissão não demonstrassem diferenças significativas quanto ao percentual de acometimento em padrão de vidro fosco entre os grupos ( Tabela 1 ). Adicionalmente, os pacientes com FAN tiveram tempo de internação hospitalar e em UTI mais prolongados que os pacientes que não apresentaram a arritmia, bem como tempo mais prolongado em ventilação mecânica invasiva ( Tabela 3 ).

No tocante ao uso de medicamentos durante internação hospitalar, para tratamento da COVID-19, houve interesse em investigar sua associação com a ocorrência de FAN. Observou-se em particular que o uso da noradrenalina, a cloroquina ou hidroxicloroquina e glicocorticoide apresentaram associação significativa com a FAN. Estes achados indicam tanto um possível efeito arritmogênico da medicação, quanto uma possível utilização compassiva naqueles pacientes com contextos clínicos de maior gravidade, mais propensos a desenvolver FAN (Tabela 3). Em particular, observamos que a amiodarona foi utilizada no grupo FAN (86,1%) e não-FAN (0,6%), com OR 1.000,00 (IC 95% [107,37; 9314,00]; p<0,001). Sete pacientes do grupo FAN necessitaram de cardioversão elétrica, com 3 pacientes (42,9%) convertendo à ritmo sinusal e, destes, dois receberam amiodarona na internação. Dois pacientes no grupo FAN fizeram CVE, não receberam amiodarona e faleceram pouco tempo depois.

Quanto às complicações clínicas observadas durante a internação, destacamos que as infecções não associadas à ventilação mecânica e o choque foram mais frequentemente observadas no grupo 1. Paradoxalmente, a distribuição relativa de disfunção ventricular esquerda entre os grupos não apresentou diferenças. Acreditamos que estes achados podem ter sido, em parte, relacionados à natureza da avaliação subjetiva da função ventricular à beira de leito, durante a internação ( Tabela 4 ). Entretanto, há necessidade de estudos com casuísticas maiores para confirmar estas observações.

Mountantonakis et al. investigando 9564 pacientes que foram internados por COVID-19, em 13 hospitais norte-americanos, observaram a presença de ritmo de FA em 1687 pacientes (17,6%). Entretanto, após excluir pacientes com história prévia de FA no momento da internação, constataram que 1109 pacientes (11,6%) apresentaram FAN. No presente trabalho, a ocorrência de FAN foi de 14,4%, sendo que os pacientes com história de FA prévia foram igualmente excluídos nessa análise. Interessantemente, comparando as ocorrências de FAN registradas em ambos os estudos, não se observa diferença significativa (respectivamente, 11,6% e 14,4%).^19^ Esta observação mostra que a estimativa encontrada no presente estudo se alinha com a literatura. Não obstante, se acrescentarmos todos os pacientes com FA aos respectivos grupos, os resultados do presente estudo ainda se alinham aos de Mountantonakis et al. (respectivamente, 19,4% e 17,6%).^19^

Notoriamente, observamos que a ocorrência da FAN esteve associada com a ventilação mecânica invasiva e a necessidade de internação em UTI, expressos pela análise dos riscos relativos, os quais foram, respectivamente, 6,67 e 20,57 ( Tabela 3 ), confirmando o contexto de gravidade clínica em que a FAN, na COVID-19, se manifesta. De fato, Mountantonakis *et al* . observaram que os pacientes com FAN tiveram maior necessidade de ventilação mecânica invasiva do que aqueles que permaneceram em ritmo sinusal (37,5% vs. 15,9%).^19^ No estudo de Pimentel et al., também se observou que a ocorrência de arritmias foi maior em pacientes em ventilação mecânica invasiva (66,7% vs 32,2%).^17^

### Modelagem prognóstica para FAN

A análise univariada de variáveis colhidas na admissão hospitalar revelou que pacientes mais idosos, com maior número de comorbidades, história de doença neurológica prévia, desorientação, história de mialgia, escore SAPS3 elevado, e baixos níveis de leucometria, plaquetometria, natremia e proteinemia são indicadores de maior risco de desenvolver FAN no contexto da pneumonia por COVID-19.

Na análise multivariada, após a dicotomização pela análise das curvas ROC, as variáveis idade > 71 anos, leucometria ≤ 7.720 cels.μL^-1^, natremia ≤ 137 mEq.L^-1^, escore SAPS3 > 55 e presença de desorientação foram as únicas preditoras independentes de FAN. Destaca-se que estas variáveis coletadas na admissão hospitalar indicam as condições clínicas de gravidade do paciente naquele momento.

A partir deste resultado, foi desenvolvido um sistema de pontuação, em que para cada variável do modelo foi atribuído o valor unitário quando ela se encontrava na faixa de gravidade, ou zero quando fora da faixa. Utilizando a curva ROC, o valor de corte ótimo do escore de gravidade para FAN > 2 apresentou uma especificidade 65,2% de sensibilidade de 82,8%. Aplicando este valor de corte e inserindo os dados em um modelo de risco proporcional de Cox, verificou-se que o HR para ocorrência de FAN foi de 7,6 ( Tabela 6 ). Este conjunto de variáveis obtidas da internação hospitalar demonstrou, assim, a eficácia de identificar aqueles pacientes em risco de FAN durante a internação.

Na literatura, encontram-se diferentes escores de risco no dimensionamento da gravidade da COVID-19. O Covid Severity Index (CSI) é baseado na avaliação de diversas variáveis e seus valores se correlacionam com a gravidade.^20^ Apesar de analisarem diversas variáveis, neste estudo os autores não analisaram a ocorrência específica de fibrilação atrial nova ou a necessidade de ventilação mecânica durante a internação, o que a nosso ver, limita os achados observados no sistema de pontuação por eles desenvolvido.

Altschul et al. chegaram a um escore de pontuação de severidade da infecção pelo SarsCoV-2, ao analisar variáveis clínicas e laboratoriais de admissão hospitalar em 4.711 pacientes hospitalizados, predizendo o risco de mortalidade. Nesta coorte observacional, os autores derivaram este escore de 2.355 pacientes e validaram em outros 2.356.^21^ A despeito do sistema daqueles autores caracterizarem a progressão da gravidade com base na agregação de condições de risco, não avaliaram a ocorrência de FAN.

Uribarri et al. avaliaram o conhecido escore prognóstico da FA, CHA_2_DS_2_-VASc, no contexto de pacientes hospitalizados por COVID-19, que desenvolveram a arritmia numa subanálise do registro HOPE ( *Health Outcome Predictive Evaluation* ) COVID-19. De um total de 6.217 pacientes, foi observada a ocorrência de FA em 250 (4.2%).^22^ Os autores empregaram um escore de uso clínico reconhecido para avaliação da gravidade no contexto da COVID-19 em pacientes com FA. Entretanto, o estudo se limitou a investigar a mortalidade associada à gravidade do escore. Não houve investigação quanto à ocorrência de FA.

Utilizando uma versão modificada do escore CHA_2_DS_2_-VASc, Abacioglu et al. investigaram pacientes internados com COVID-19, e constataram que a taxa de mortalidade hospitalar foi proporcional à gravidade, definida pela pontuação do escore.^23^ Este estudo reforça a utilidade dos sistemas de pontuação disponíveis na prática clínica para estimar a gravidade de pacientes no momento da admissão hospitalar. Entretanto, prescindiram da menção ao risco de desenvolvimento de FAN.

Por se tratar de uma doença infecciosa nova com atividade pró-inflamatória e pró-trombótica, faz-se necessária uma avaliação objetiva e quantitativa do risco de desenvolver uma arritmia como a fibrilação atrial, que sabidamente aumenta o risco trombogênico. Com base na revisão da literatura, identificamos, no melhor do nosso conhecimento, que este é o primeiro trabalho a elaborar um sistema de pontuação baseado em informações clínicas e laboratoriais, avaliadas no momento da admissão hospitalar, com a finalidade de avaliar o risco de ocorrência de FAN em pacientes internados com pneumonia por COVID-19.

A análise *post-hoc* das variáveis independentes do modelo de Cox indica que os quantitativos dos grupos foram satisfatórios para atingir adequada potência estatística ([1 – β] > 85% para idade, SAPS3 e escore FAN, para o erro alfa de 0,05). Entretanto, novos estudos com casuísticas maiores são necessários para validar estes resultados.

### Limitações

As informações clínicas, epidemiológicas, laboratoriais obtidas dos prontuários eletrônicos dos pacientes foram avaliadas retrospectivamente. As buscas no prontuário médico foram planejadas e sistematizadas para a identificação dos eventos de FAN durante a internação hospitalar. Foi utilizado para este fim um algoritmo simplificado para identificação de um conjunto de termos, que representavam a fibrilação atrial (por exemplo: “fibrilação”+“atrial”, “fib”+“atrial”, “fib”+“atrail”, etc.). A despeito da verificação redundante das informações do prontuário, é possível que alguns registros, que não utilizaram termos usuais, possam não ter sido detectados. Não obstante, os achados nesse trabalho se mostram semelhantes aos observados na literatura com casuísticas mais amplas, o que indica que os dados tiveram boa qualidade.

As informações relativas ao ritmo cardíaco foram obtidas de registros do prontuário médico digital. A confirmação do ritmo cardíaco nos registros eletrocardiográficos foi feita em 80% dos pacientes. Em cerca de 1/5 dos prontuários eletrônicos analisados, a documentação do ritmo cardíaco foi feita exclusivamente com base na descrição da evolução médica durante a internação, que pode ser uma limitação.

Diversos sintomas foram avaliados na admissão hospitalar (tabela 2), todavia o sintoma “palpitação” não foi relatado com frequência. Devido à gravidade da apresentação clínica da maioria dos pacientes, a pesquisa por palpitação não se constituiu de forma sistemática relevante, o que pode ser considerado como uma limitação.

O fato de os pacientes internados em UTI apresentarem maior ocorrência de FAN poderia residir no fato de estarem continuamente monitorados em relação aos internados em enfermaria, tendo em vista que a arritmia pode ocorrer de forma assintomática. Este fato também poderia ser uma limitação.

Na avaliação do modelo de risco proporcional de Cox, dos 201 pacientes, apenas 135 apresentavam dados completos da variável SAPS3 (89% do grupo 1 e 68,5% do grupo 2) para realizar as análises, o que é uma limitação. Entretanto, a potência estatística elevada atingida na análise *post-hoc* desta variável indica que os resultados foram relevantes. Para o cálculo do escore FAN, foram usadas as estratégias de melhor e de pior cenários, substituindo os valores atribuídos ao SAPS3 de, respectivamente, 0 e 1, aos valores faltantes. A distribuição de valores do escore FAN > 2 nos grupos 1 e 2 foram, respectivamente, 82,8% e 34,6%, em ambos os cenários, o que indica que o impacto foi limitado neste estudo.

## Conclusão

A fibrilação atrial nova (FAN) é uma arritmia comum em pacientes com 60 anos ou mais, hospitalizados por COVID-19 e pneumonia viral, correspondendo a 14,4 % das internações.

A FAN está relacionada à apresentação clínica e laboratorial de maior gravidade na admissão hospitalar, à internação em UTI e à ventilação mecânica invasiva.

Na admissão hospitalar, idade maior que 71 anos, leucometria global menor que 7.720 cels.μL-¹, natremia inferior a 137 mEq.L^-1^, presença de desorientação e escore SAPS3 com valor acima de 55 constituem um conjunto de marcadores prognósticos independentes para FAN.
