# Supplementary material for: Variáveis Clínicas e Laboratoriais na Admissão Hospitalar são Preditores de Fibrilação Atrial Nova em Pacientes Internados com Pneumonia por COVID-19
Source: Arq Bras Cardiol. 2024 Mar 20;121(1):e20220784. [Article in Portuguese] doi: 10.36660/abc.20220784 (PMC11149983; doi:10.36660/abc.20220784)
Supplement: Translation

# Translation: Clinical and Laboratory Data Up on Hospital Admission are Predictors of New-Onset Atrial Fibrillation in Patients Hospitalized Due to COVID-19 Pneumonia

**Authors:** Bruno Rustum Andrea, Paulo Roberto Benchimol-Barbosa, Simone Farah, Alexandra Monteiro

**Affiliations:** 1 Rio de Janeiro RJ Brazil Hospital Universitário Pedro Ernesto - Telemedicina e Telessaúde da Faculdade de Ciências Médicas, Rio de Janeiro, RJ – Brazil; 2 Rio de Janeiro RJ Brazil Hospital Universitário Pedro Ernesto - Coordenadoria de Medicina Clínica, Rio de Janeiro, RJ – Brazil

**Keywords:** COVID-19, Atrial Fibrillation, Prognosis, Hospitalization, Predictive Value of Tests

## Abstract

**Background:**

New-onset atrial fibrillation (NOAF) occurs in patients hospitalized due to COVID-19. It is still unknown whether clinical and laboratory data assessed upon hospital admission have predictive value for NOAF.

**Objectives:**

To analyze, upon hospital admission, variables with predictive potential for the occurrence of NOAF in patients with COVID-19 pneumonia.

**Methods:**

Observational, retrospective, case-control study. Electronic medical reports of consecutive patients, 60 years of age or older, hospitalized due to COVID-19 pneumonia between March 1st and July 15^th^, 2020, were reviewed. Non-paired Student or chi-squared tests compared variables. A Cox proportional hazard model was employed to identify independent predictors of NOAF. P value < 0.05 was considered statistically significant.

**Results:**

Among 667 patients hospitalized due to COVID-19, 201 (30.1%) fulfilled the inclusion criteria. NOAF was documented in 29 patients (14.4%), composing group 1. Group 2 was composed of 162 patients without NOAF. Ten patients were excluded due to the AF rhythm upon hospital admission. In groups 1 and 2, there were differences in overall in-hospital survival rate (24.1 % vs. 67.9%; p<0.001), length of stay in ICU (11.1 ± 10.5 days vs. 4.9 ± 7.5 days; p=0.004) and need for mechanical ventilation rate (82.9% vs. 32.7%; p<0.001). In the Cox model, age > 71 y/o (HR=6.8; p<0.001), total leukocyte count ≤ 7,720 cels.μL-¹ (HR=6.6; p<0.001), serum [Na+] ≤ 137 mEq.L-¹ (HR=5.0; p=0.001), SAPS3 score > 55 (HR=5.6; p=0.002), and disorientation (HR=2.5; p=0.04) on admission were independent predictors of NOAF.

**Conclusion:**

NOAF is a common arrhythmia in elderly hospitalized patients with COVID-19 pneumonia. Clinical and laboratory parameters evaluated on admission have a predictive value for the occurrence of NOAF during hospitalization.

## Introduction

The pandemic of the disease caused by the novel coronavirus that began in late 2019 (COVID-19) with its epicenter in the city of Wuhan, China, quickly spread around the world, bringing catastrophic consequences to public health and the global economy.^1^

Characterd SARS-CoV-2, the new coronavirus predisposes to an initial viral pneumonia, but the main clinical consequence stems from a widespread systemic inflammatory reaction.^2^The major clinical manifestations are pulmonary and cardiovascular, and individuals with a worse prognosis are those who are older and previously had heart disease.^3 , 4^

Atrial fibrillation (AF) is a common cardiac arrhythmia in people over 55 years old^5^and is often triggered in patients with inflammatory clinical contexts such as myocarditis.^6^

In COVID-19, the acute occurrence of AF seems to be associated with a systemic inflammatory state.^7^Several studies show the relationship between COVID-19 and new-onset AF (NOAF), suggesting that the acute manifestation of the arrhythmia is associated with a reserved prognosis.^8^At the time of hospital admission, many patients present with severe respiratory conditions, characterized by intense dyspnea and low arterial oxygen saturation, associated with viral pneumonia in chest computed tomography, reflecting an advanced systemic inflammatory condition.

Therefore, in this scenario, clinical, epidemiological, and laboratory information evaluated at hospital admission may have prognostic value for the development of NOAF during hospitalization.

This study aimed to investigate the predictive value of clinical, epidemiological, and laboratory variables obtained at hospital admission for the development of NOAF in elderly patients hospitalized with COVID-19 pneumonia.

## Methods

### Study design

This is an observational, quantitative, longitudinal, case-control study with a retrospective analysis of data from electronic medical records prospectively collected between March 1st and July 15th, 2020, from a cohort of consecutive patients hospitalized at the Pedro Ernesto University Hospital (HUPE), State University of Rio de Janeiro, diagnosed with COVID-19.

The Research Ethics Committee approved this study under number CAAE 35192920.2.0000.528, and the use of informed consent was dispensed.

### Analyzed Variables

The variables analyzed in this study were collected upon the patient's hospital admission.

Hospital admission was defined as the first complete assessment within the first 24 hours of admission, including medical evaluation, laboratory tests, and imaging exams. The date of hospital admission was defined as the date when the patient was admitted to the COVID-19 unit.

Admitted patients had three main origins: i – patients coming from the municipal or state regulation system with suspected or confirmed COVID-19; ii – patients coming from their own homes for screening at the hospital; and iii – patients hospitalized for other reasons who developed the infection during hospitalization.

Admitted patients were placed in intensive care units or wards specially developed for their care.

#### Demographic Variables

The analyzed demographic variables were age and gender.

#### Clinical Comorbidities

Information regarding clinical comorbidities was extracted using a text keyword search and their variations and abbreviations, using an active search algorithm including spaces, specially developed for this purpose. This data was collected and is available in the Red Cap database at the Medical Sciences School of the State University of Rio de Janeiro. To ensure the reliability of information, the data was triple-checked by a specialist for quality control.

The clinical comorbidities collected and analyzed were systemic arterial hypertension (SAH), diabetes mellitus (DM), coronary artery disease (CAD), chronic kidney disease (CKD), chronic obstructive pulmonary disease (COPD), asthma, obesity, and body mass index (BMI), smoking, neoplasia, chronic liver disease, autoimmune disease, immunologic disease, chronic hematologic disease, chronic neurological disease, prior heart failure (HF). The total number of comorbidities was also evaluated for each patient.

### Medications

Some previously used medications suggested to relate to COVID-19 pathophysiological mechanisms were analyzed, such as angiotensin-converting enzyme inhibitor (ACEI) or angiotensin II receptor blocker (ARB),^9^metformin,^10^statins,^11^and non-steroidal anti-inflammatory drugs (NSAID).

### Signs and Symptoms at Hospital Admission

Signs and symptoms were collected from the patient's medical history upon admission. These variables included fever, cough, dysosmia, dysgeusia, asthenia, rhinorrhea, dyspnea, disorientation, agitation or drowsiness, odynophagia, myalgia, diarrhea, nausea, vomiting, headache, syncope, hypotension, and oxygen saturation (SpO_2_) below 94%.

Hypotension was characterized as systolic blood pressure below 100 mmHg. Dyspnea was a subjective complaint reported in the medical record by the patient or family members. The variable disorientation was defined using the Glasgow scale of less than 15, as described in medical records.

### Clinical-hospital parameters

The following information was also collected for analysis during hospitalization: date of admission to the intensive care unit (ICU), need for invasive mechanical ventilation, occurrence of shock due to any cause, and need for renal replacement therapy. Additionally, the durations in days from the onset of symptoms to the date of hospital admission, total hospitalization, ICU stay, admission to the start of mechanical ventilation, total mechanical ventilation duration, and admission to the first episode of AF were collected.

The severity scores of SOFA (Sequential Organ Failure Assessment)^12^and SAPS3 (Simplified Acute Physiology Score 3)^13^were evaluated upon hospital admission.

The Lawton scale was used to assess the clinical frailty of the elderly, as it evaluates instrumental activities of daily living.^14^

### Laboratory variables

Laboratory tests evaluated upon the patient's hospital admission were: D-dimer, lactate dehydrogenase, hematocrit, hemoglobin, leukogram, lymphocyte count, platelet count, oxaloacetic and pyruvic transaminase, urea, creatinine, sodium, potassium, ferritin, troponin I, triglycerides, total cholesterol and high and low-density fractions, fibrinogen, international normalized ratio (INR), precursor of brain natriuretic peptide (NT-pro-BNP), total bilirubin, glucose, total proteins, plasma albumin, creatine kinase, glomerular filtration rate by CKD-EPI.

#### Imaging Exams

##### Chest Computed Tomography

Chest computed tomography (CT) exams were performed on the day of hospital admission or the following day, and the images were available for online consultation. The devices used were Brilliance 64 channels (Philips, Netherlands), SOMATOM Scope 16 channels (Siemens Healthcare GmbH, Germany), and Revolution ACT 16 channels (General Electrics, USA).

Some patients were transferred to HUPE with a chest CT report performed at the original hospital. In these cases, the information from the report was obtained from the medical records.

The lung patterns on chest CT used to classify patients with COVID-19-associated viral pneumonia were described by Mogami et al. and were characterized by the typical pattern of densely dotted opacities, typically distributed peripherally in the lung regions, generally designated as “ground-glass opacities”.^15^

In this study, the severity of pulmonary manifestation was characterized by the percentage of lung parenchyma area affected with a “ground-glass” pattern. Two-dimensional tomographic reconstruction was analyzed from the apex to the base in sections with thickness ranging from 3 to 5 mm.

The calculation of the total affected area in ground glass was done by outlining the percentage of involvement of each tomographic section, summed up across all sections. This sum was later divided by the number of sections, thus obtaining the average percentage of pulmonary involvement.

For the analysis, dichotomization was arbitrarily defined at the 50% threshold as ≥ 50% and < 50%.

##### Echocardiogram

Evaluation of cardiac cavity dimensions and right and left ventricular function was performed using the InnoSight™ portable ultrasound equipment (Phillips, Netherlands) through the “point of care ultrasound” (POCUS) protocol.

The echocardiographic evaluations performed at the bedside by POCUS were made through subjective assessment of cavity dimensions, segmental contractility, and global systolic function of the left ventricle (LV).

The presence of left ventricular (LV) dysfunction was defined when the bedside transthoracic echocardiogram by POCUS demonstrated characteristic alterations (global or segmental hypocontractility of the LV walls) or when NT-pro-BNP was elevated.

As many patients did not have quantitative registration of ejection fraction, we decided to use the available qualitative analysis of ventricular function (ventricular dysfunction).

### Data extraction

The data were extracted from the standardized electronic medical record system (MVPEP), and the initial criterion used for patient identification was to be admitted to the HUPE in the COVID-19 unit with a confirmed diagnosis by RT-PCR.

Data was extracted from medical records for analysis from March 1st, 2020, to July 15th, 2020.

Initially collected data included: medical record number, full name, first admission unit, attendance number (hospital admission authorization code – AIH), age, gender, ethnicity, admission date, discharge/death date, movement between units during hospitalization with respective admission dates in each unit, RT-PCR test date, and its result.

For the selection of patients with NOAF, it was necessary to record the occurrence of sinus rhythm or regular rhythm upon admission and atrial fibrillation rhythm during hospitalization.

The occurrence of NOAF was identified considering the following information in the medical records: i – registration of medical progress in the electronic medical record with serial evaluation of heart rhythm; ii – descriptive reports of electrocardiograms (ECG) in the electronic medical record, and; iii – visualization of ECG tracings.

In case the printed ECG register was not available in the medical records, the occurrence of NOAF was considered when it was reported on the event date and in subsequent medical progress notes.

### Inclusion and exclusion criteria

All patients in this study had only one hospitalization in the evaluated period.

A reviewing medical committee composed of specialists from Pedro Ernesto University Hospital was established to evaluate all electronic medical records of patients hospitalized with a presumptive diagnosis of COVID-19 until July 15th, 2020. The committee consisted of a geriatrician, a rheumatologist, a pulmonologist, and an infectious disease specialist.

The inclusion criteria for this study were:

- Age of 60 years or older.
- Confirmed molecular diagnosis of COVID-19 by RT-PCR.
- Imaging diagnosis of COVID-19 viral pneumonia.
- In patients who had NOAF, a documentation of at least one episode of AF during hospitalization was necessary.

Patients were excluded if:

- They were younger than 60 years old.
- There was no confirmed molecular diagnosis of COVID-19.
- Chest CT not available.
- Documentation of AF rhythm upon hospital admission.

### Clinical Outcome: New-Onset Atrial Fibrillation (NOAF)

For prognostic evaluation, NOAF was treated as the primary outcome.

### Statistical analyses

#### Variable comparison

Continuous variables were presented as mean ± standard deviation (SD), and categorical variables as percentages or ratios. Patients who developed NOAF were designated as group 1, and those who did not develop the arrhythmia formed group 2. The probability distribution of the variables was evaluated using the Pearson skewness test. Variables with an absolute value greater than 3 were considered non-normal and, therefore, log-transformed for statistical comparison to normalize probability distributions. Normally distributed numerical variables were compared between groups using an unpaired Student's t-test. The comparison of variances for appropriate application of the t-test was performed using Levene's test or Snedecor's F-test, when necessary. Categorical variables were compared using the chi-square test or Fisher's exact test. Relative risk (RR) or odds ratio (OR) and their respective 95% confidence intervals (CI) were calculated when appropriate.

The applications used for statistical analyses were Medcalc v. 10.3.2 (MedCalc Software Ltd, Belgium) and Microsoft Excel 2021 and 365 (Microsoft Corporation, Redmond, Washington, USA) with XRealStats supplement (Real Statistics Using Excel. https://www.real-statistics.com/, last accessed on 06/10/2022).

#### Statistical modeling

ROC curve analysis was used for the dichotomization of numerical variables and to define the optimal cut-off value. To investigate the prognostic value of selected variables with the occurrence of NOAF, the Cox proportional hazards model was used, considering a one-variable rule in the model for every five NOAF outcomes. After Cox analysis for NOAF outcome, Kaplan-Meier curves of significant variables were generated.

It was of interest to evaluate the variables obtained at admission as indicators of in-hospital progression until the development of NOAF. Therefore, uni- and multivariate analyses were used, considering the time from hospital admission to the occurrence of NOAF. Significant variables in the univariate model were entered into a multivariate model to identify independent predictors of NOAF outcome. The independent predictor variables were used to develop a scoring system to assess the risk of NOAF. For this purpose, the beta coefficients of significant variables from the regression equation were rounded to the nearest integer. Thus, in the scoring system, each independent variable had two assignable values: zero or the value obtained by rounding its beta coefficient. Considering the composition of the scoring system as the sum of the values attributed to each variable, the minimum possible value was zero, and the maximum was the sum of the values attributed to each variable.

Post-hoc analysis was performed to calculate the statistical power (1 - β) achieved in this study, using the chi-square test for comparisons between groups 1 and 2. The ratios of the quantifications of each group were used to calculate the value of the achieved power. The alpha error level was set at 0.05 for all statistical tests.

## Results

From March 1st to July 15th, 2020, 667 patients were hospitalized due to COVID-19 after evaluation by the review committee. Among these, 201 (30.1%) met the inclusion criteria for this study ( Figure 1 ). Atrial fibrillation (AF) was documented in 39 patients (19.4%), with 10 individuals excluded from the analysis for presenting AF at the time of admission. Thus, 29 patients (14.4%) were classified as group 1, and 162 patients who did not develop AF formed group 2. One patient in group 1 had only a record of atrial flutter on the ECG and was included in the analysis. The mean time of AF occurrence after hospital admission was 13.7 ± 14.0 days, and the total hospitalization time in groups 1 and 2 was 30.5 ± 27.6 days and 16.2 ± 12.4 days, respectively. In group 1, 89.7% of the patients were in sinus rhythm at the end of hospitalization. Thus, AF was associated with a more severe context, and an overview of the natural history evolution of these patients is presented on the timeline (Central Illustration).

**Figure 1:** Flowchart for patient inclusion.

Regarding clinical and demographic data, individuals in group 1 were older, had a higher prevalence of chronic neurological disease, and had a greater number of comorbidities compared to group 2 ( Table 1 ).

**Table 1:** Epidemiological and clinical characteristics of the population upon hospitalization

Variables	NOAF (n=29)	Non-NOAF (n=162)	p value	RR for NOAF
Age (years) *	73.9 ± 8.5	69.8 ± 7.4	0.008 †	
Female (n (%))	8 (27.6)	69 (42.6)	0.189 ‡	0.56 CI 95% [0.26 – 1.19]
Systemic arterial hypertension (n (%))	24 (82.8)	118 (72.8)	0.326 ‡	1.56 CI 95% [0.63 – 3.86]
Diabetes mellitus (n (%))	11 (37.9)	73 (45.1)	0.574 ‡	0.75 CI 95% [0.38 – 1.50]
Coronary artery disease (n (%))	8 (28.6)	28 (17.6)	0.248 ‡	1.55 CI 95% [0.74 – 3.23]
Chronic renal disease (n (%))	5 (17.2)	24 (14.8)	0.738 ‡	1.09 CI 95% [0.45 – 2.62]
Chronic obstructive pulmonary disease (n (%))	4 (13.8)	15 (9.3)	0.647 ‡	1.38 CI 95% [0.54 – 3.53]
Asthma (n (%))	0 (0.0)	2 (1.2)	0.924 ‡	N/A
Obesity (n (%))	8 (30.8)	35 (26.7)	0.755 ‡	1.13 CI 95% [0.53 – 2.40]
BMI (kg/m^2^)*	28.1 ± 14.2	28.4 ± 14.7	0.674 §	
Smoking (n (%))	8 (29.6)	48 (31.4)	0.868 ‡	0.88 CI 95% [0.41 – 1.90]
Neoplasia (n (%))	2 (6.9)	27 (16.9)	0.264 ‡	0.38 CI 95% [0.10 – 1.52]
Chronic liver disease (n (%))	3 (10.3)	10 (6.3)	0.640 ‡	1.45 CI 95% [0.51 – 4.16]
Autoimmune diseases (n (%))	2 (7.1)	4 (2.5)	0.484 ‡	2.32 CI 95% [0.71 – 7.60]
Immunological diseases (n (%))	3 (10.3)	12 (7.5)	0.780 ‡	1.25 CI 95% [0.43 – 3.67]
Chronic hematological disease (n (%))	1 (3.4)	5 (3.1)	0.635 ‡	1.10 CI 95% [0.18 – 6.81]
Chronic neurological disease (n (%))	9 (31.0)	16 (9.9)	0.005 ‡	2.87 CI 95% [1.48 – 5.59]
Previous heart failure (n (%))	8 (27.6)	21 (12.9)	0.067 ‡	1.82 CI 95% [0.89 – 3.70]
Other comorbidities (n (%)) †	18 (64.3)	75 (48.7)	0.145 ‡	1.57 CI 95% [0.77 – 3.21]
Number of comorbidities before admission *	4.1 ± 1.4	3.4 ± 1.7	0.005 ¶	
ACEI/ARB (n (%))	18 (62.1)	94 (58.0)	0.608 ‡	1.08 CI 95% [0.54 – 2.15]
Metformin (n (%))	5 (17.2)	31 (19.1)	0.994 ‡	0.87 CI 95% [0.36 – 2.13]
Simvastatin (n (%))	11 (37.9)	58 (36.0)	0.709 ‡	1.01 CI 95% [0.51 – 2.02]
NSAID (n (%))	2 (8.0)	5 (3.3)	0.533^‡^	1.83 CI 95% [0.53 – 6.26]
Number of medications before admission *	3.6 ± 2.6	3.1 ± 3.0	0.186 ¶	
Lawton scale before admission *	15.8 ± 9.0	18.0 ± 9.3	0.099 ¶	
SOFA score before admission *	5.6 ± 4.2	4.4 ± 3.9	0.013 ¶	
SAPS3 score before admission *	60.6 ± 28.1	54.9 ± 29.1	0.037 ¶	
Lung involvement >=50% upon admission (n (%))	16 (55.2)	78 (48.1)	0.483 ‡	1.18 CI 95% [0.06 – 2.32] †

ACEI: Angiotensin-Converting Enzyme Inhibitors; ARB: Angiotensin II Receptor Antagonists; NSAID: Non-steroidal Anti-Inflammatory Drugs; BMI: body mass index; NOAF: New-Onset Atrial Fibrillation.

* mean ± SD.

† Other comorbidities included alcoholism, ex-smoking, Alzheimer's disease, rheumatoid arthritis and vasculitis, cerebrovascular disease, dementia, depression, dyslipidemia, gout, hypothyroidism, osteoporosis, peripheral arterial disease, aortic aneurysm, history of deep vein thrombosis.

‡ Comparison using chi-square test.

§ Comparison using unpaired Student's t-test.

¶ Comparison using the Mann-Whitney test.

Concerning symptoms recorded at hospital admission, group 1 showed higher frequencies of myalgia and disorientation. Other common COVID-19 symptoms did not differ between groups ( Table 2 ).

**Table 2:** Characteristics of symptoms in the population before hospitalization

Variables	NOAF (n=29)	Non-NOAF (n=162)	p value	RR for NOAF
Fever (n (%))	23 (79.3)	108 (66.7)	0.243	1.69 CI 95% [0.73 – 3.94]
Cough (n (%))	15 (51.7)	111 (68.5)	0.115	0.53 CI 95% [0.27 – 1.03]
Dysosmia (n (%))	4 (13.8)	12 (7.4)	0.436	1.75 CI 95% [0.70 – 4.40]
Dysgeusia (n (%))	1 (3.4)	6 (3.7)	0.778	0.82 CI 95% [0.13 – 5.18]
Asthenia (n (%))	11 (37.9)	70 (43.2)	0.689	0.80 CI 95% [0.40 – 1.60]
Myalgia (n (%))	13 (44.8)	37 (22.8)	0.025	2.20 CI 95% [1.14 – 4.25]
Rhinorrhea (n (%))	4 (13.8)	15 (9.3)	0.679	1.45 CI 95% [0.56 – 3.72]
Dyspnea (n (%))	28 (96.6)	141 (87.0)	0.227	3.46 CI 95% [0.50 – 24.19]
Oxygen Saturation < 94% (n (%))	27 (93.1)	147 (90.7)	0.836	1.25 CI 95% [0.33 – 4.82]
Disorientation (n (%))	13 (44.8)	35 (21.6)	0.016	2.28 CI 95% [1.18 – 4.38]
Agitation/Drowsiness pre-hospitalization (n (%))	9 (31.0)	42 (25.9)	0.626	1.17 CI 95% [0.57 – 2.39]
Odynophagia (n (%))	1 (3.4)	9 (5.6)	0.981	0.64 CI 95% [0.10 – 4.26]
Diarrhea (n (%))	1 (3.4)	30 (18.5)	0.081	0.18 CI 95% [0.03 – 1.26]
Nausea (n (%))	1 (3.4)	13 (8.0)	0.628	0.45 CI 95% [0.07 – 3.08]
Vomiting (n (%))	1 (3.4)	13 (8.0)	0.628	0.45 CI 95% [0.07 – 3.08]
Headache (n (%))	2 (6.9)	17 (10.5)	0.795	0.67 CI 95% [0.17 – 2.60]
Syncope (n (%))	2 (6.9)	6 (3.7)	0.779	1.69 CI 95% [0.48 – 5.88]
Hypotension (n (%))	1 (3.4)	13 (8.0)	0.623	0.45 CI 95% [0.07 – 3.06]

NOAF: New-Onset Atrial Fibrillation; RR: Relative Risk.

Regarding severity indicators evaluated at hospital admission, patients in group 1 showed significantly higher SOFA and SAPS3 scores than group 2 ( Table 1 ). There were no differences between groups in the distribution of lung involvement above 50% upon admission ( Table 1 ).

Additionally, concerning patients’ hospital evolution, as shown in Table 3 , group 1 patients had a higher need for ICU admission and invasive mechanical ventilation use, as well as a longer ICU stay and total invasive mechanical ventilation time. There were no differences in the need for renal replacement therapy, lung involvement above 50%, and the time from symptom onset to hospital admission. The use of medications such as norepinephrine, glucocorticoids, chloroquine, and hydroxychloroquine was significantly higher in group 1. There was no statistical significance in the association between the use of other evaluated medications and the occurrence of AF ( Table 3 ).

**Table 3:** Clinical and hospital parameters and pharmacological therapies used during hospitalization

Variables	NOAF (n=29)	Non-NOAF (n=162)	p value	OR for NOAF
CIU admission (n (%))	28 (96.6)	80 (49.4)	<0.001	27.01 CI 95% [3.59 – 203.28]
Mechanical ventilation (n (%))	24 (82.8)	53 (32.7)	<0.001	90.2 CI 95% [3.26 – 24.96]
Hemodialysis (n (%))	10 (34.5)	32 (19.8)	0.116	1.90 CI 95% [0.81 – 4.48]
Time from onset of symptoms to date of hospital admission (days) *	7.6 ± 4.3	8.5 ± 5.1	0.380	
Total length of hospital stay (days) *	30.5 ± 27.6	16.2 ± 12.4	0.010	
Length of stay in ICU (days) *	11.1 ± 10.5	4.9 ± 7.5	0.004	
Time from hospital stay to start of mechanical ventilation (days) *	7.6 ± 9.2	4.2 ± 5.1	0.101	
Length in mechanical ventilation (days) *	12.9 ± 14.0	9.5 ± 5.9	0.008	
Time from hospitalization to the first episode of AF (days) *	13.7 ± 14.0	-	-	
Norepinephrine (n (%))	27 (93.1)	60 (37.0)	<0.001	22.95 CI 95% [5.27 – 99.95]
Chloroquine / Hydroxychloroquine (n (%))	13 (44.8)	42 (25.9)	0.042	2.32 CI 95% [1.03 – 5.23]
Glucocorticoid use (n (%))	23 (79.3)	66 (40.7)	<0.001	5.58 CI 95% [2.15 – 14.44]
Heparin (unfractionated or low molecular weight) (n (%))	26 (89.7)	139 (86.5)	0.628	1.37 CI 95% [0.38 – 4.92]
Ivermectin (n (%))	11 (37.9)	44 (27.2)	0.241	1.64 CI 95% [0.72 – 3.74]

ICU: Intensive Care Unit; NOAF: New-Onset Atrial Fibrillation; OR: Odds Ratio.

* mean ± SD.

There were no differences in the occurrence of intra-hospital complications analyzed between groups 1 and 2, except for the rates of non-pulmonary nosocomial infection and shock, which were significantly higher in group 1 ( Table 4 ). Hospital mortality rates in patients from group 1 and group 2 were 75.9% vs. 32.1%, with an RR for death of 2.36 ( Table 4 ).

**Table 4:** Analysis of the odds ratios of in-hospital clinical complications (above) and analysis of the relative risk of death (below) in the NOAF and Non-NOAF groups

Variables	NOAF (n=29)	Non-NOAF (n=162)	p value	OR for NOAF
Disseminated intravascular coagulation (n (%))	0 (0)	1 (0,6)	0,327	
Deep vein thrombosis (n (%))	1 (3,6)	5 (3,1)	0,653	
Pulmonary thromboembolism (n (%))	0 (0)	3 (1,9)	0,654	
Hospital pulmonary infection (n (%))	15 (51,7)	62 (39,2)	0,259	
Hospital infection of any non-pulmonary nature (n (%))	13 (44,8)	24 (14,8)	<0,001	4,67 CI 95% [1,79 – 6,40]
Delirium (n (%))	6 (20,7)	42 (25,9)	0,579	
Left ventricular dysfunction (n (%))	16 (55,2)	90 (55,6)	0,842	0,83 CI 95% [0,37 – 1,84]
Shock (n (%))	18 (62,1)	44 (27,2)	<0,001	4,39 CI 95% [1,92 – 10,03]

OR: Odds Ratio; NOAF: New-Onset Atrial Fibrillation.

Intra-hospital laboratory variables were compared between groups and are shown in Table 5 . Group 1 presented lower values in leukocyte count, platelet count, AST, serum sodium, and total serum proteins. Moreover, the percentage of patients with troponin I values > 0.2 ng/mL was higher in group 1.

**Table 5:** Laboratory tests

Variables †	NOAF (n=29)	Non-NOAF (n=162)	p value	RR for NOAF
D-dimer (ng.mL^-1^) †	6181.9 ± 15112.2	5120.4 ± 11443.3	0.723	
LDH (U.L^-1^) †	383.0 ± 194.4	448.8 ± 251.7	0.199	
HTO (%) †	36.3 ± 5.7	35.8 ± 6.9	0.736	
HGB (g.dL^-1^) †	12.1 ± 1.9	11.8 ± 2.5	0.469	
WBC (cels.μL^-1^) †	6726.2 ± 2894.0	9482.4 ± 10398.1	0.005	2.63 IC 95% [1.18–5.86]
LYNPH (%) †	13.7 ± 8.4	15.4 ± 11.0	0.418	
PLA (cels.μL^-1^) †	188.1 ± 96.9	252.4 ± 116.7	0.006	4.15 IC 95% [1.94–8.88]
GOT (U.L^-1^) †	38.4 ± 19.6	59.0 ± 111.8	0.036	2.45 IC 95% [1.20–5.02]
GPT (U.L^-1^) † *	30.3 ± 15.2	43.4 ± 56.8	0.224	
GGT (U.L^-1^) † *	107.4 ± 86.6	139.2 ± 168.8	0.193	
URE (mg.dL^-1^) †	60.5 ± 48.0	60.8 ± 49.3	0.979	
FER (pmol.L^-1^) † *	1611.2 ± 1225.8	2005.7 ± 3087.3	0.328	
CRE (mg.dL^-1^) †	1.4 ± 1.0	1.8 ± 2.0	0.162	
Tn I (>0,2 ng.mL^-1^)	30.2 %	12.2 %	0.038	2.46 IC 95% [1.07–5.69]
FIB (mg.dL^-1^) †	537.8 ± 270.8	480.5 ± 200.3	0.264	
INR (AU) †	1.3 ± 0.2	1.2 ± 0.2	0.111	
NT-pro-BNP (pg.mL^-1^) †	3884.0 ± 8619.2	5172.2 ± 8659.4	0.602	
PTN C R (mg.L^-1^) †	85.1 ± 88.6	58.2 ± 52.6	0.128	
BAST (absolute count) †	92.0 ± 254.1	181.2 ± 657.3	0.205	
PTT (seconds) †	31.3 ± 9.9	29.3 ± 5.9	0.300	
K (mEq.L^-1^) †	4.5 ± 0.9	4.4 ± 0.8	0.538	
NA (mEq.L^-1^) †	136.7 ± 5.9	139.8 ± 6.8	0.019	2.51 IC 95% [1.26–5.01]
BIL (mg.dL^-1^) †	0.7 ± 0.4	0.7 ± 0.8	0.560	
GLU (mg.dL^-1^) †	171.5 ± 111.8	156.5 ± 120.1	0.576	
PTN (g.dL^-1^) †	5.9 ± 0.7	6.4 ± 1.4	0.042	2.75 IC 95% [1.33–5.68]
ALB (g.dL^-1^) †	2.8 ± 0.6	3.1 ± 0.6	0.089	
CPK (U.L^-1^) †	179.5 ± 243.5	223.3 ± 528.8	0.526	
TRI (mg.dL^-1^) † *	140.2 ± 51.0	156.4 ± 76.0	0.751	
COL (mg.dL^-1^) †	150.2 ± 38.3	144.7 ± 46.3	0.578	
HDL (mg.dL^-1^)) † *	32.0 ± 6.4	34.9 ± 13.7	0.947	
LDL (mg.dL^-1^) †	93.2 ± 32.2	86.5 ± 41.2	0.459	
GFR CKD-EP (ml/min) †	79.0 ± 20.8	82.8 ± 25.7	0.460	

ALB: plasma albumin; BAST: absolute bat count; BIL: total bilirubin; CRE: creatinine; COL: total cholesterol; CPK: creatine phosphokinase; FER: ferritin; FIB: fibrinogen; GLU: blood glucose; GGT: gamma-glutamyl transferase; HGB: hemoglobin; HDL: High-Density Lipoprotein; HTO: hematocrit; K: potassium; LDH: lactic dehydrogenase; LDL: Low-Density Lipoprotein; WBC: white blood cell count; LINF: relative lymphocyte count; NA: Natremia; NT-pro-BNP: precursor of brain natriuretic peptide; PLA: platelet count; PTN: total proteins; CRPtn: C-reactive protein; PTT: partial thromboplastin time; INR: international standardized relationship; GFR CKD-EPI: glomerular filtration rate by CKD-EPI; GOT: Glutamic-oxaloacetic transaminase; GPT: Glutamic-pyruvic transaminase; TnI: troponin I; TRI: triglycerides; URE: urea.

* Mann-Whitney comparative test.

† mean ± SD.

### Prognostic analysis and modeling for AF

The ROC curve analysis defined the cut-off values for the variables as follows: age > 71 years (sensitivity 69%, specificity 62.1%), number of comorbidities > 4 (sensitivity 48.3%, specificity 77.0%), SAPS3 > 55 (sensitivity 75.0%, specificity 77.7%), total leukocyte count ≤ 7,720 cells/μL (sensitivity 75.9%, specificity 49.7%), platelets < 196,000 cells/μL (sensitivity 72.4%, specificity 67.7%), AST < 37 U/L (sensitivity 64.3%, specificity 62.1%), serum sodium < 137 mEq/L (sensitivity 62.1%, specificity 64.4%), total proteins < 6 g/dL (sensitivity 66.7%, specificity 64.5%). These dichotomized variables, as well as significant categorical variables, were analyzed through the univariate Cox proportional hazard model, with results presented in Table 6 .

**Table 6:** Univariate and multivariate analysis of predictors of the occurrence of NOAF by Cox proportional regression (above) and an optimal cut-off value of the risk assessment score for the occurrence of NOAF (below)*

	Univariate Analysis			Univariate Analysis			
	β	HR CI 95%	p value	β	HR CI 95%	p value	Score
Age > 71 years	1.200	3.3 [1.5 – 7.3]	0.003	1.913	6.8 [2.5 – 18.3]	<0.001	1
Number of comorbidities > 4	0.840	2.3 [1.1 – 4.8]	0.024				
Neurological disease	1.150	3.2 [1.4 – 7.0]	0.005				
Disorientation	1.179	3.3 [1.5 – 6.9]	0.002	0.919	2.5 [1.05 – 6.0]	0.04	1
Myalgia	1.007	2.7 [1.3 – 5.7]	0.007				
Nosocomial infection	1.025	2.8 [1.3 – 5.8]	0.007				
Shock	1.178	3.2 [1.5 – 7.0]	0.003				
Mechanical ventilation	1.902	6.7 [2.6 – 17.5]	<0.001				
ICU admission	2.891	18.0 [8.5 – 131.5]	0.005				
Troponin > 0,2 ng.mL^-1^	0.651	1.9 [0.8 – 4.8]	0.170				
Noradrenaline use	2.622	13.8 [3.3 – 57.8]	<0.001				
SAPS3 > 55	1.173	3.2 [1.3 – 8.1]	0.013	1.723	5.6 [1.9 – 16.6]	0.002	1
Total leukocyte count ≤ 7.720 cels.μL^-1^	1.170	3.2 [1.4 – 7.6]	0.008	1.887	6.6 [2.2 – 19.9]	<0.001	1
Platelets < 196.000 cels.μL^-1^	1.503	4.5 [2.0 – 10.1]	<0.001				
GOT < 37 U.L^-1^	0.986	2.7 [1.2 – 5.8]	0.014				
Natremia < 137 mEq.L^-1^	0.836	2.3 [1.1 – 4.9]	0.030	1.611	5.0 [1.9 – 13.1]	0.001	1
Total proteins < 6 g.dL^-1^	1.247	3.5 [1.5 – 8.0]	0.004				
Optimal cut-off value	Specificity (%) §	Sensitivity(%) §	AUC §	p-value †	HR ‡ §		
> 2	65.2 [57.3 – 72.5]	82.8 [64.2 – 94.1]	0.815 [0.717 – 0.913]	<0.001	7.6 [2.9 – 19.8]		

HR: hazard ratio; CI: Confidence interval; NOAF: New Onset Atrial Fibrillation; GOT: Glutamic-oxaloacetic transaminase;

* N of group 1 = 29; N of group 2 = 162;

† Cox function adjusted p-value < 0,001; χ^2^15,0; r^2^=0,927;

‡ N=135;

§ [CI 95].

In the multivariate analysis of the Cox proportional hazard model, employing variables with a significant p-value, it was demonstrated that age > 71 years, leukocyte count < 7,720 cells/μL, serum sodium < 137 mEq/L, SAPS3 > 55, and disorientation were independent predictors for the occurrence of AF during hospitalization ( Table 6 , Figure 2 ).

**Figure 2:** Kaplan-Meier curves of the probability of NOAF-free survival of the variables collected at hospital admission that are independent predictors of NOAF (analyzed by the Cox multivariate model) and the Kaplan-Meier curve of the NOAF score: A – Age; B – Global leukometry; C – Serum sodium; D – SAPS3 score; E – Disorientation; F – NOAF score. New Onset Atrial Fibrillation (NOAF); SAPS3 – Simplified Acute Physiology Score III.

Based on the result of the multivariate Cox model, a scoring system was developed to assess the risk of AF occurrence in these patients. Thus, according to the beta values of the variables, scores of 1 were assigned to each of the variables when in the severity range or zero when outside this range. This score resulted in 6 possible scores: 0 to 5 ( Table 6 ). The distribution of patients according to the score was: 0 points = 8.9%; 1 point = 26.7%; 2 points = 30.4%; 3 points = 25.2%; 4 points = 5.2%; 5 points = 3.7%. Applying ROC curve analysis to the score distribution, the optimal cut-off point was > 2 with high specifitity and sensitivity (table 6). Using the Cox proportional hazard model, the HR for a cut-off value > 2 for AF was 7.6 ( Table 6 and Figure 2 ).

### Post-Hoc analysis of sample statistical power

Post-hoc sample analysis was performed regarding the distributions of independent predictor variables between groups 1 and 2, considering an alpha error of 0.05. The statistical power (1 - β) achieved for the AF outcomes of the variable age > 71 years was 89%, total leukocyte count ≤ 7,720 cells/μL was 73%, serum sodium ≤ 137 mEq/L was 76%, disorientation was 73%, and SAPS3 > 55 was 92%. Regarding the AF score, considering the distribution of values > 2 in groups 1 and 2 of 82.8% and 34.6%, respectively, the post-hoc analysis revealed statistical power > 99%.

## Discussion

In the present study, new-onset atrial fibrillation (AF) was shown to be a frequent event in hospitalized patients with COVID-19 pneumonia, observed in 14.4% of patients in sinus rhythm at admission. In 2021, a global survey showed that the occurrence of AF and atrial flutter in the general population with COVID-19 was 9.0%, being more common in Europe (21.9%).^16^A Brazilian study showed that the presence of arrhythmias in 241 consecutive patients hospitalized with COVID-19 was 8.7%, with 76.2% being atrial arrhythmias.^17^Additionally, in our study, 26 out of 29 patients who had AF (89.7%) were in sinus or regular rhythm at the end of hospitalization, indicating that AF was a transient clinical phenomenon in most patients. Furthermore, AF was associated with a context of greater severity when evaluated by clinical and laboratory indices from hospital admission (Central Illustration).

Indeed, Pardo Sans et al. evidenced that AF was associated with a context of greater severity and reserved prognosis in a cohort of 160 consecutive patients hospitalized for COVID-19.^18^However, these authors added patients who had previous AF to the group that did not develop new-onset AF and compared it with the group that developed AF. In our work, we chose to standardize the groups, excluding patients with previous AF from the analysis. Thus, two homogeneous groups were constituted in the context of atrial arrhythmia.

Regarding demographic variables, previous comorbidities, and symptoms recorded at the time of admission, patients’ age, chronic neurological disease, total number of comorbidities, disorientation, and myalgia had a significant impact on the univariate analysis for the occurrence of AF during hospitalization ( Table 2 ).

Patients who presented AF had characteristics of greater severity at hospital admission, as evaluated by SOFA and SAPS3 scores when compared to patients who did not present AF ( Table 1 ). These conditions were reflected in more frequent ICU admissions and a higher need for invasive mechanical ventilation ( Table 3 ). However, the pulmonary changes in the chest CT scan at admission did not show significant differences in the percentage of ground-glass opacities between the groups ( Table 1 ). Additionally, patients with AF had a longer length of hospital and ICU stay than patients who did not present the arrhythmia, as well as a longer duration of invasive mechanical ventilation ( Table 3 ).

Regarding the use of medications during hospitalization for COVID-19 treatment, there was interest in investigating their association with the occurrence of AF. It was observed, in particular, that the use of noradrenaline, chloroquine or hydroxychloroquine, and glucocorticoids showed a significant association with AF. These findings indicate both a possible arrhythmogenic effect of the medication and a possible compassionate use in those patients with more severe clinical contexts, more prone to developing AF ( Table 3 ). Particularly, we observed that amiodarone was used in the AF group (86.1%) and non-AF group (0.6%), with OR 1,000.00 (95% CI [107.37; 9314.00]; p<0.001). Seven patients from the AF group required electrical cardioversion, with 3 patients (42.9%) converting to sinus rhythm, and of these, two received amiodarone during hospitalization. Two patients in the AF group underwent electrical cardioversion, did not receive amiodarone, and died shortly afterward.

Regarding clinical complications observed during hospitalization, we highlight that infections not associated with mechanical ventilation and shock were more frequently observed in group 1. Paradoxically, the relative distribution of left ventricular dysfunction between the groups did not show differences. We believe that these findings may have been partly related to the subjective nature of assessing ventricular function at the bedside during hospitalization ( Table 4 ). However, larger case studies are needed to confirm these observations.

Mountantonakis et al., investigating 9,564 patients hospitalized for COVID-19 in 13 North American hospitals, observed the presence of AF rhythm in 1,687 patients (17.6%). However, after excluding patients with a previous history of AF at the time of admission, they found that 1,109 patients (11.6%) had new-onset AF. In the present study, the occurrence of new-onset AF was 14.4%, and patients with a history of previous AF were equally excluded from this analysis. Interestingly, comparing the occurrences of new-onset AF recorded in both studies, no significant difference was observed (respectively, 11.6% and 14.4%).^19^This observation shows that the estimate found in the present study aligns with the literature. Nevertheless, if we add all patients with AF to their respective groups, the results of the present study still align with those of Mountantonakis et al. (respectively, 19.4% and 17.6%).^19^

Notably, we observed that the occurrence of new-onset AF was associated with invasive mechanical ventilation and the need for ICU admission, as expressed by the analysis of relative risks, which were, respectively, 6.67 and 20.57 (table 3), confirming the clinical severity context in which NOAF manifests in COVID-19. Indeed, Mountantonakis et al. observed that patients with NOAF had a greater need for invasive mechanical ventilation than those who remained in sinus rhythm (37.5% vs. 15.9%).^19^Pimentel et al. also observed that the occurrence of arrhythmias was higher in patients on invasive mechanical ventilation (66.7% vs. 32.2%).^17^

### Prognostic modeling for new-onset atrial fibrillation

The univariate analysis of variables collected at hospital admission revealed that older patients, those with a higher number of comorbidities, a history of previous neurological disease, disorientation, a history of myalgia, elevated SAPS3 score, and low levels of leukocytes, platelets, serum sodium, and serum protein are indicators of a higher risk of developing new-onset AF in the context of COVID-19 pneumonia.

In the multivariate analysis, after dichotomization by ROC curve analysis, the variables age > 71 years, leukocyte count ≤ 7,720 cells/μL, sodium ≤ 137 mEq/L, SAPS3 score > 55, and presence of disorientation were the only independent predictors of new-onset AF. It is noteworthy that these variables collected at hospital admission indicate the patient's clinical severity conditions at that time.

Based on this result, a scoring system was developed in which a unit value was assigned for each variable of the model when it was in the severity range or zero when outside the range. Using the ROC curve, the optimal cut-off value of the severity score for new-onset AF > 2 showed a specificity of 65.2% and sensitivity of 82.8%. By applying this cut-off value and then admitting the data into a Cox proportional hazards model, it was found that the HR for the occurrence of new-onset AF was 7.6 ( Table 6 ). This set of variables obtained from hospitalization thus demonstrated the effectiveness of identifying those patients at risk of new-onset AF during hospitalization.

In the literature, different risk scores are found in assessing the severity of COVID-19. The COVID Severity Index (CSI) is based on the assessment of various variables, and their values correlate with severity.^20^Despite analyzing various variables, in this study, the authors did not analyze the specific occurrence of new-onset atrial fibrillation or the need for mechanical ventilation during hospitalization, which, in our view, limits the findings observed in the scoring system they developed.

Alschul et al. arrived at a severity scoring system for SARS-CoV-2 infection by analyzing clinical and laboratory variables at hospital admission in 4,711 hospitalized patients, predicting the risk of mortality. In this observational cohort, the authors derived this score from 2,355 patients and validated it in another 2,356.^21^Despite characterizing the system of those authors in terms of the progression of severity based on the aggregation of risk conditions, they did not evaluate the occurrence of new-onset AF.

Uribarri et al. evaluated the well-known prognostic score for AF, CHA_2_DS_2_-VASc, in the context of hospitalized patients with COVID-19 who developed the arrhythmia in a sub-analysis of the HOPE (Health Outcome Predictive Evaluation) COVID-19 registry. Out of a total of 6,217 patients, the occurrence of AF was observed in 250 (4.2%).^22^The authors employed a clinically recognized score for assessing severity in the context of COVID-19 in patients with AF. However, the study was limited to investigating mortality associated with the severity of the score. There was no investigation into the occurrence of AF.

Using a modified version of the CHA_2_DS_2_-VASc score, Abacioglu et al. investigated patients hospitalized with COVID-19 and found that the hospital mortality rate was proportional to the severity defined by the score.^23^This study reinforces the usefulness of scoring systems available in clinical practice to estimate the severity of patients at the time of hospital admission. However, they did not mention the risk of developing new-onset AF.

Because it is a new infectious disease with pro-inflammatory and pro-thrombotic activity, an objective and quantitative evaluation of the risk of developing an arrhythmia, such as atrial fibrillation, known to increase thrombotic risk, is necessary. Based on the literature review, to the best of our knowledge, we identified that this is the first work to develop a scoring system based on clinical and laboratory information evaluated at the time of hospital admission, aimed at assessing the risk of new-onset AF occurrence in patients hospitalized with COVID-19 pneumonia.

In the present study, the post-hoc analysis of the independent variables of the Cox model indicates that the sample size of the groups was satisfactory to achieve adequate statistical power ([1 - β] > 85% for age, SAPS3, and AF score, for an alpha error of 0.05). However, further studies with larger case numbers are needed to validate these results.

### Limitations

The clinical, epidemiological, and laboratory information obtained from patients’ electronic medical records was retrospectively assessed. The search in the medical records was planned and systematized to identify incidents of new-onset atrial fibrillation (AF) during hospitalization. A simplified algorithm was used to identify a set of terms representing atrial fibrillation (for example: “fibrillation” + “atrial,” “fib” + “atrial,” “fib” + “atrail,” etc.). Despite the redundant verification of the medical records, some records that did not use common terms might not have been detected. However, the findings in this study were similar to those observed in the literature with larger case studies, indicating good data quality.

Information regarding cardiac rhythm was obtained from digital medical record entries. Cardiac rhythm confirmation in electrocardiographic records was conducted in 80% of the patients. In about 1/5 of the analyzed electronic records, documentation of cardiac rhythm was exclusively based on medical progress notes during hospitalization, which could be a limitation.

Several symptoms were evaluated at hospital admission ( Table 2 ); however, the symptom “palpitation” was not frequently reported. Due to the severity of the clinical presentation in most patients, the investigation for palpitations did not systematically constitute a relevant factor, which could be considered a limitation.

The higher occurrence of AF in ICU-admitted patients might be due to continuous monitoring compared to those admitted to regular wards, as arrhythmia can occur asymptomatically. This factor could also be a limitation.

In the evaluation of the Cox proportional hazard model, out of 201 patients, only 135 had complete SAPS3 variable data (89% from group 1 and 68.5% from group 2) for analysis, which is a limitation. However, the high statistical power achieved in the post-hoc analysis of this variable indicates the relevance of the results. For the calculation of the AF score, best and worst-case strategies were used, replacing missing SAPS3 values with 0 and 1, respectively. The distribution of AF score values > 2 in groups 1 and 2 were 82.8% and 34.6%, respectively, in both scenarios, indicating limited impact in this study.

## Conclusion

Atrial fibrillation new onset (AF) is a common arrhythmia in patients aged 60 years or older hospitalized for COVID-19 and viral pneumonia, accounting for 14.4% of admissions.

AF is associated with a more severe clinical and laboratory presentation upon hospital admission, ICU admission, and invasive mechanical ventilation.

Upon hospital admission, age greater than 71 years, total leukocyte count less than 7,720 cells/μL, serum sodium lower than 137 mEq/L, SAPS3 score above 55, and presence of disorientation constitute a set of independent prognostic markers for AF.
